# Isomeric Effects of Au_28_(S-*c*-C_6_H_11_)_20_ Nanoclusters on Photoluminescence: Roles of Electron-Vibration Coupling and Higher Triplet State

**DOI:** 10.1021/acsnano.4c06702

**Published:** 2024-08-02

**Authors:** Abhrojyoti Mazumder, Kang Li, Zhongyu Liu, Yitong Wang, Yong Pei, Linda A. Peteanu, Rongchao Jin

**Affiliations:** † Department of Chemistry, Carnegie Mellon University, Pittsburgh, Pennsylvania 15213, United States; ‡ Department of Chemistry, Key Laboratory of Environmentally Friendly Chemistry and Applications of MOE, Xiangtan University, Xiangtan, Hunan 411105, China

**Keywords:** atomically precise gold nanoclusters, structural isomers, near-infrared photoluminescence, cryogenic spectroscopy, higher-lying triplet states, nonradiative decay

## Abstract

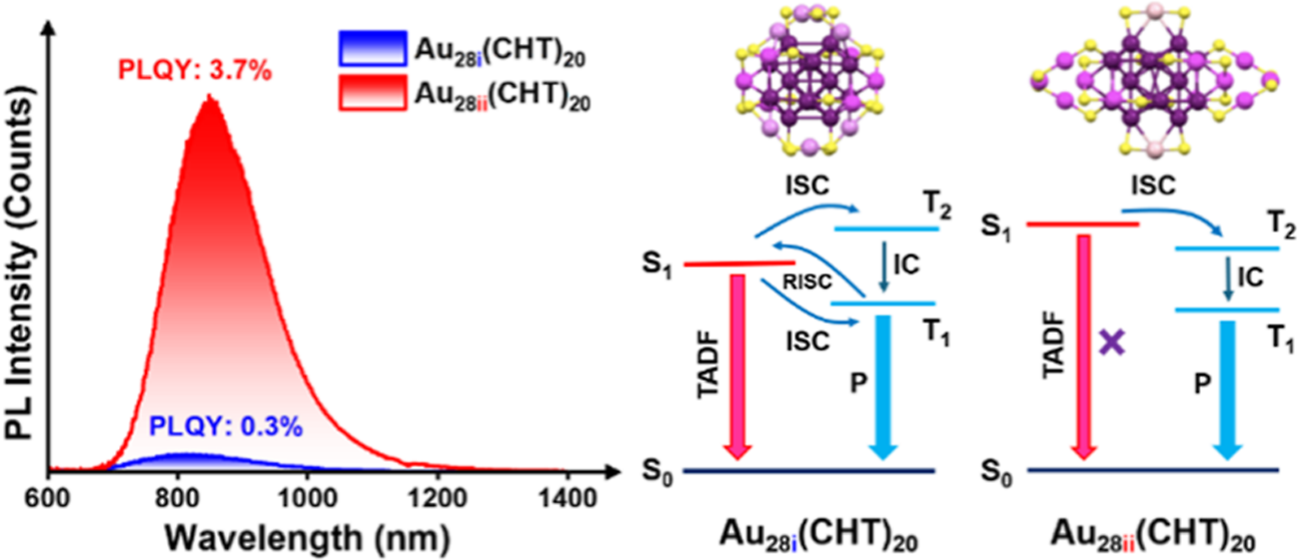

The exploration of near-infrared photoluminescence (PL) from atomically precise nanoclusters is currently a prominent area of interest owing to its importance in both fundamental research and diverse applications. In this work, we investigate the near-infrared (NIR) photoluminescence mechanisms of two structural isomers of atomically precise gold nanoclusters of 28 atoms protected by cyclohexanethiolate (CHT) ligands, i.e., Au_28i_(CHT)_20_ and Au_28ii_(CHT)_20_. Based on their structures, analysis of ^3^O_2_ (triplet oxygen) quenching of the nanocluster triplet states, temperature-dependent photophysical studies, and theoretical calculations, we have elucidated the intricate processes governing the photoluminescence of these isomeric nanoclusters. For Au_28i_(CHT)_20_, its emission characteristics are identified as phosphorescence plus thermally activated delayed fluorescence (TADF) with a PL quantum yield (PLQY) of 0.3% in dichloromethane under ambient conditions. In contrast, the Au_28ii_(CHT)_20_ isomer exhibits exclusive phosphorescence with a PLQY of 3.7% in dichloromethane under ambient conditions. Theoretical simulations reveal a larger singlet (S_1_)–triplet (T_1_) gap in Au_28ii_ than that in Au_28i_, and the higher T_2_ state plays a critical role in both isomers’ photophysical processes. The insights derived from this investigation not only contribute to a more profound comprehension of the fundamental principles underlying the photoluminescence of atomically precise gold nanoclusters but also provide avenues for tailoring their optical properties for diverse applications.

## Introduction

Near-infrared photoluminescence is one of the pivotal properties of atomically precise metal nanoclusters (NCs), fueled by the dual objectives of unraveling the PL mechanism and exploring luminescent NCs for applications in diverse fields, such as biomedical imaging/sensing.^1−11^ Despite the advancements to date, most metal NCs exhibit low PL quantum yields in the NIR (e.g., a few percent or lower), necessitating critical emphasis on their NIR PL enhancement.^12−15^ Various strategies, such as aggregation-induced emission, ligand engineering, intracluster interaction, staple motif tailoring, and heteroatom doping, have been reported with mechanistic insights;^16−23^ however, the detailed PL mechanisms still remain elusive in many cases. Thus, exploring NIR PL and understanding the mechanisms are an ongoing effort.

From the structural point of view, deciphering the underlying PL mechanisms for isomeric NCs (e.g., the same core but different surface motifs) can offer deep insights into the structural factors that affect the PL. The importance of structure–property correlations is evident in fundamental studies. While structural isomerism has been well studied in molecules,^24^ it remains challenging in nanomaterials^25^ due to the lack of atomic precision and detailed structures. Recent efforts in creating atomically precise, isomeric NCs show great promise in achieving the fundamental structure–property correlations for the PL.^26−29^

The quasi-isomerism phenomenon in thiolate (SR)-protected Au_28_(SR)_20_ (i.e., different R groups) was observed in 2016, and stable structures were obtained with cyclohexanethiolate (abbrev. CHT) and *p*-*tert*-butylbenzenethiolate (abbrev. TBBT) as the protecting ligands.^30^ Single-crystal X-ray diffraction (SCXRD) revealed that both structures of Au_28_(SR)_20_ (SR = CHT and TBBT) share the same core (i.e., a four-tetrahedral Au_14_ core) but have differences in the surface staple motifs. Specifically, the Au_28_(CHT)_20_ is protected by four Au_3_(SR)_4_ trimers and two Au(SR)_2_ monomers, whereas Au_28_(TBBT)_20_ is protected by two Au_3_(SR)_4_ trimers and four Au_2_(SR)_3_ dimers.^30^ The study on the PL–structure correlation in Au_28_(SR)_20_ (CHT vs TBBT) was carried out in 2020 by Chen et al., who found that Au_28_(CHT)_20_ was ∼15-fold higher in photoluminescence quantum yields (PLQY) than Au_28_(TBBT)_20_.^31^

In 2020, Wu’s group observed an intriguing structural oscillation in Au_28_(CHT)_20_ and discovered true isomerism in Au_28_(CHT)_20_, named Au_28i_(CHT)_20_ and Au_28ii_(CHT)_20_.^26^ They obtained the structure of Au_28ii_(CHT)_20_ from SCXRD, which was same as that of Au_28_(CHT)_20_ reported by Chen et al.,^31^ but no good crystal for Au_28i_(CHT)_20_ was obtained; rather, they obtained good crystals of CPT (CPT = cyclopentanethiolate)-protected Au_28_, i.e., Au_28_(CPT)_20_, which had the same structure as that of Au_28_(TBBT)_20_. They found that the calculated UV–vis–NIR spectrum of Au_28i_ by assuming the structure of the CPT-protected one was consistent with the Au_28i_ experimental spectrum; thus, Au_28i_ was believed to adopt a similar structure as that of Au_28_(CPT)_20_ despite the CPT versus CHT ligand difference. Interestingly, in these two isomers, Au_28ii_ was found to be more luminescent under ambient conditions than both Au_28i_ and Au_28_(TBBT)_20_,^26^ which implied that the surface motifs played a significant role in the PL. From the structural determination, Wu’s group reported that the trimeric staple should contribute more to the emission intensity compared with the dimeric staple and the monomeric staple due to the increasing rigidity, which can reduce the low-energy motion and thus increase the PL emission. In a related work, Pei’s group recently performed density functional theory (DFT) simulations to unravel the PL origin in the Au_28_(CHT)_20_ and Au_28_(TBBT)_20_ quasi-isomers.^27^

Despite the observation of different PL values in the two Au_28_(CHT)_20_ isomers, there has been no in-depth investigation into the complex PL mechanism. This knowledge gap prompted our interest to delve deeper and establish a comprehensive PL mechanism in this isomeric system, particularly because the roles of the core and staple motifs in isomeric NCs can be separated and studies on the NIR emission in isomeric NCs are still scarce.

In this study, we investigate the PL mechanisms of the isomeric Au_28_(CHT)_20_ NCs by performing time-resolved PL, cryogenic spectroscopies, triplet state probing, and theoretical calculations. Both experiment and simulations reveal an effective reverse intersystem crossing (ISC) (T_1_ to S_1_) in the Au_28i_ isomer and accordingly concurrent TADF and phosphorescence emission, but the Au_28ii_ isomer emits phosphorescence only due to the suppressed T_1_ to S_1_ conversion. In temperature-dependent studies, although Au_28ii_ is more emissive than Au_28i_ at room temperature, the vibration-induced nonradiative decay in Au_28i_ is more efficiently suppressed at 80 K, making Au_28i_ significantly surpass the PL intensity of Au_28ii_, the latter only showing a modest enhancement as its low-frequency vibrations are not effectively suppressed at 80 K. DFT simulations show that the low and high frequency vibrations are associated with the long and short staples, respectively. The obtained PL and structure correlations will be helpful for enhancing the PL in other NCs.

## Results/Discussion

**Figure 1:**
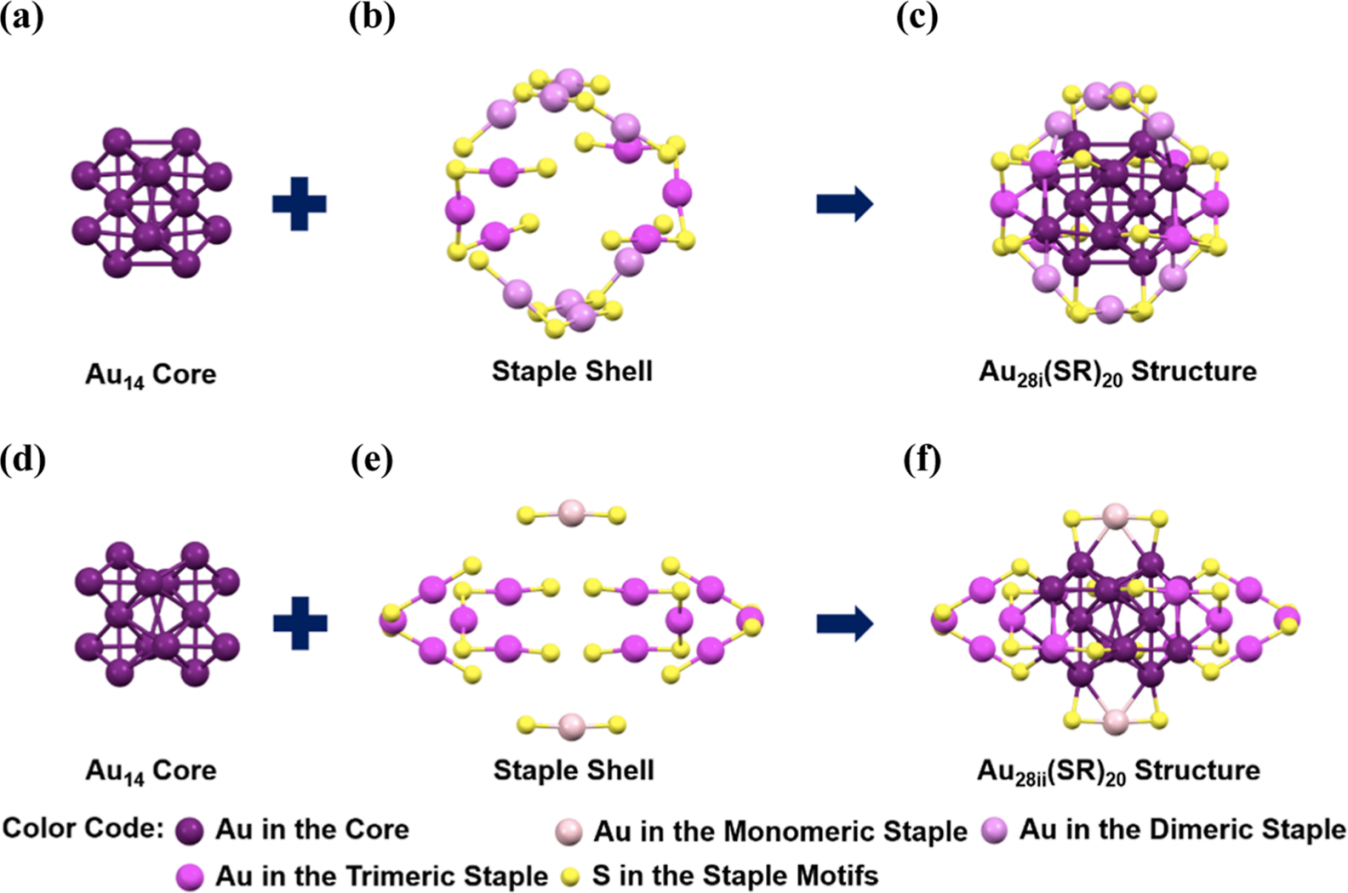
X-ray structures of isomeric **Au**_**28i**_**(CHT)**_**20**_ (panels a–c) and **Au**_**28ii**_**(CHT)**_**20**_ (d–f). Carbon tails are omitted for clarity.

Previous work by Wu’s group has established the structures of the two Au_28_(CHT)_20_ isomers by SCXRD.^26^ Both possess the same Au_14_ core (with an average shortest Au–Au bond distance of ∼2.8 Å), but each has a different arrangement of staple motifs. Specifically, the shell of Au_28i_ comprises two trimers and four dimers (Figure 1a–c), whereas the shell of Au_28ii_ comprises four trimers and two monomers (Figure 1d–f). We followed the synthesis reported by Wu’s group and prepared the isomeric NCs. The chromatography-purified NCs were then used in the PL studies, as discussed below.

### Photophysical Studies of the Isomeric Au_28_(CHT)_20_ NCs under Ambient Conditions

**Figure 2:**
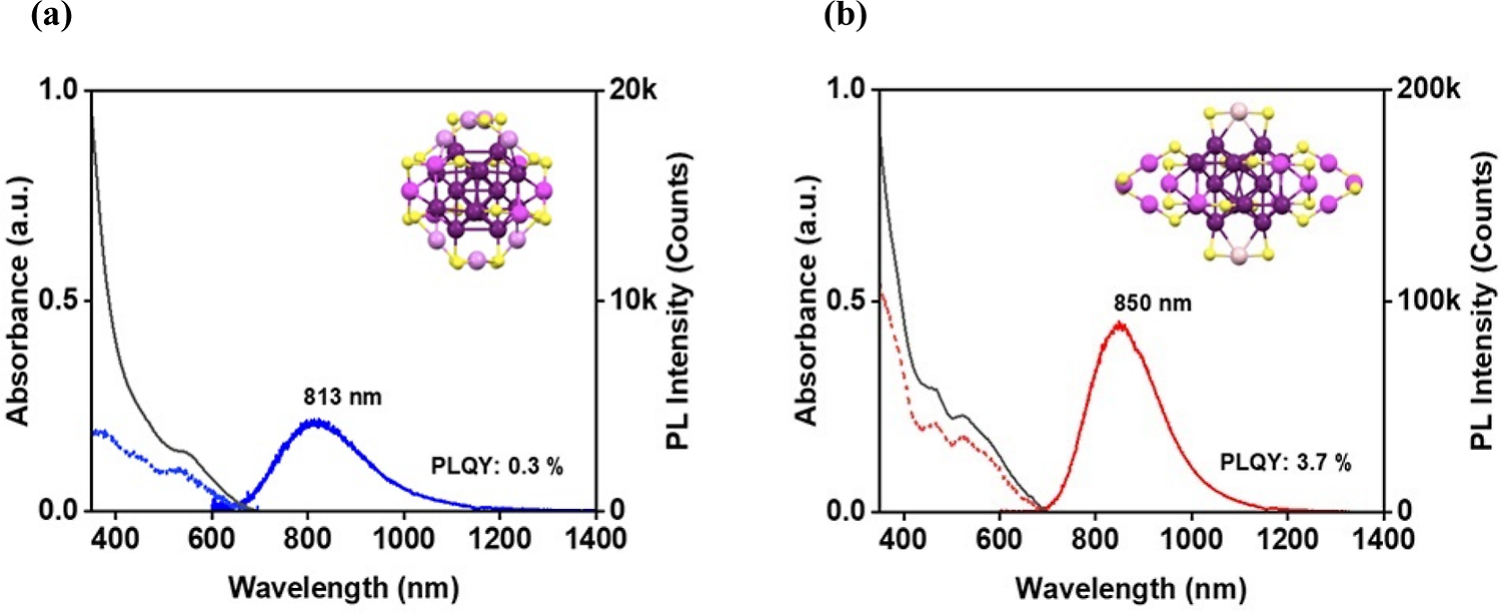
(a,b) Optical absorption spectra (black lines; absorbance shown on the left *y*-axes), PL spectra (solid colored lines; intensities on the right *y*-axis), and excitation spectra (dotted lines; intensities on the right *y*-axis) of **Au**_**28i**_**(CHT)**_**20**_ and **Au**_**28ii**_**(CHT)**_**20**_, respectively. Other conditions: NCs in DCM, under ambient conditions; slit width for PL measurements: 5/5 nm, and for PLE measurements: 8/8 nm. PL spectra were collected at 0.7 optical density at 365 nm (the excitation wavelength).

The optical absorption and photoluminescence spectra of the two isomeric Au_28_(CHT)_20_ NCs in diluted dichloromethane (DCM) solutions were examined under ambient conditions, as illustrated in Figure 2. The Au_28i_ NC (Figure 2a) displays absorption bands at 446 and 545 nm, whereas the Au_28ii_ NC (Figure 2b) exhibits absorption bands primarily at 468 and 518 nm, and a shoulder at 580 nm is also found. Both NCs show almost the same optical gap (*E*_g_) of 1.8 eV (Figure S1 inset).

The two isomers display near-infrared PL centered at 813 (1.53 eV) for Au_28i_ and 850 (1.46 eV) for Au_28ii_ upon excitation at 365 nm (3.40 eV). The agreement between the absorption and PL excitation (PLE) profiles (Figure 2, dashed lines) affirms that the luminophores are the Au_28_(SR)_20_ NCs, rather than any impurity, and that the PL originates from the *E*_g_ gap in the core. The PLQY were determined by a relative method using the Au_25_ rod as a standard (its PL at 900 nm and PLQY ∼8% under ambient conditions in solution).^32^ The difference in PLQY (∼11.6-fold enhancement from Au_28i_ to Au_28ii_) at room temperature suggests a notable role of staple motifs (Figure 1b vs e) in the PLQY since their cores are the same. The presence of more trimeric staples in Au_28ii_, compared to dimeric and monomeric staples, should contribute to the higher PLQY, which is likely due to the increased rigidity.^26^

**Figure 3:**
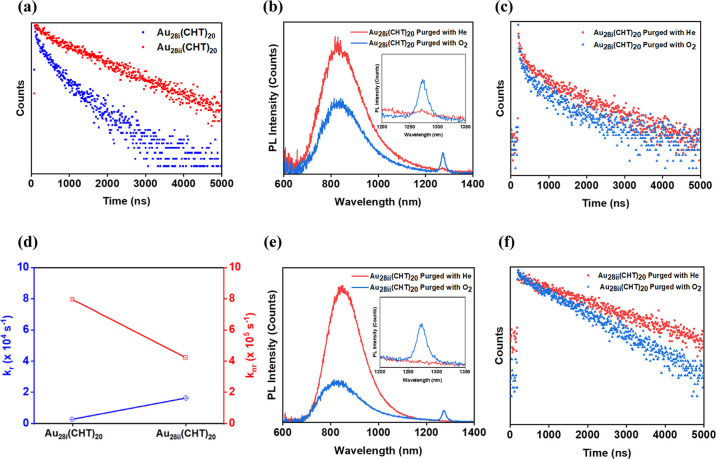
(a) PL decay profiles of the isomeric **Au**_**28**_**(SR)**_**20**_ NCs under ambient conditions in DCM. (b,c) PL spectra and decay profiles of **Au**_**28i**_ in deuterochloroform under a helium atmosphere and O_2_ atmosphere, respectively (excitation: 365 nm). (d) Plot of radiative decay rate constant (blue symbol) and nonradiative decay rate constant (red symbol) of the isomeric **Au**_**28**_**(SR)**_**20**_ NCs under ambient conditions in DCM. (e,f) PL spectra and decay profiles of **Au**_**28ii**_ in deuterochloroform under helium and O_2_ atmosphere, respectively (excitation: 365 nm).

The PL lifetimes were determined by time-correlated single photon counting (TCSPC), which also exhibits a distinct difference between Au_28i_ and Au_28ii_ (Figure 3a) at room temperature under ambient conditions. Au_28i_ shows two lifetime components: 168 ns (10.6%, τ_1_) and 1379 ns (89.4%, τ_2_) (average τ_av_: 1251 ns), note that the percentage in the parentheses indicates the relative amplitude of the component, whereas Au_28ii_ showed only one component (τ: 2281 ns) (Table 1); note that the lifetime of Au_28ii_ is consistent with the 1.7 μs from previous nanosecond transient absorption measurements,^31^ and the ultrafast 11 ps component in previous femtosecond transient absorption measurements pertains to structural relaxation.^31^ The results signify different relaxation pathways in Au_28i_ and Au_28ii_. The radiative rate constant (*k*_r_) and nonradiative rate constant (*k*_nr_) for the two NCs were calculated. For Au_28i_, *k*_r_ ∼ 0.3 × 10^4^ s^–1^ and *k*_nr_ ∼ 8.0 × 10^5^ s^–1^, and for Au_28ii_*k*_r_ ∼ 1.6 × 10^4^ s^–1^ and *k*_nr_ ∼ 4.2 × 10^5^ s^–1^ (Table 1). The significantly higher (5-fold) *k*_r_ in Au_28ii_ along with a lower nonradiative relaxation rate by a factor of two both makes Au_28ii_ more luminescent. This also correlates with the greater flexibility in Au_28i_ staple motifs giving rise to the higher nonradiative rate due to the vibrational energy loss through the staple motifs. The comparison of their *k*_r_ and *k*_nr_ suggests that the QY enhancement in Au_28ii_ with more trimeric staple motifs is mainly associated with the suppression of nonradiative relaxation and the enhancement of radiative relaxation (Figure 3d).

**Table 1:** Photophysical Data for the Isomeric Au_28_(SR)_20_ NCs in DCM Solution under Ambient Conditions^a^

nanoclusters	Au_28i_	Au_28ii_
Φ_PL_ (%)	0.3	3.7
lifetime (ns)	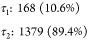	τ: 2281 (100%)
*k*_r_ (s^–1^)^b^	0.3 × 10^4^	1.6 × 10^4^
*k*_nr_ (s^–1^)^c^	8.0 × 10^5^	4.2 × 10^5^
*E*_g_ (eV)	1.8	1.8
PL peak (eV)	1.53	1.46

a The PL lifetimes are extracted from Figure 3a.

b Calculated by *k*_r_ = Φ_PL_·τ_av_^–1^.

c Calculated by *k*_nr_ = (1 – Φ_PL_)·τ_av_^–1^.

We further tested the PL sensitivity of the two isomeric Au_28_(SR)_20_ NCs (dissolved in deuterochloroform) to O_2_. Deuterochloroform exhibits weaker NIR absorption (i.e., vibrational overtones) compared to other solvents, which can reduce solvent absorption-induced distortion of the NIR PL spectra.^12^ The overall integrated intensity of PL was reduced in both NCs under pure O_2_ compared to He, and the appearance of singlet oxygen (^1^O_2_) PL signal at 1272 nm (a sharp phosphorescence peak) can be readily observed in both NCs (Figure 3b,e insets), implying the existence of triplet state population in both NCs and their sensitization of triplet oxygen (the ground state of O_2_) to singlet oxygen (the excited state). The triplet state population of the isomeric Au_28_(SR)_20_ NCs implies that their emission can be phosphorescence and/or TADF^16,33−36^ because both types of PL are originated from the population in the triplet excited state (T_1_). In the deuterochloroform solution of Au_28i_, its average PL lifetime (τ_av_) is 1320 ns [components τ_1_ = 95 ns (8.1%) and τ_2_ = 1428 ns (91.9%)] under He and is decreased to 960 ns [component τ_1_ = 80 ns (11.7%) and τ_2_ = 1077 ns (88.3%)] under O_2_ atmosphere (Figure 3c). In the case of Au_28ii_(CHT)_20_, its sole PL lifetime τ = 2447 ns (100%) under He is decreased to 1588 ns (100%) under the O_2_ atmosphere (Figure 3f).

In regard to the two lifetime components in Au_28i_, τ_1_ can be tentatively assigned as fluorescence as it did not change much from the He to O_2_ atmosphere, whereas the lifetime τ_2_ should be the triplet-state emission due to its microsecond timescale and sensitivity to O_2_. For the case of Au_28ii_, its only long lifetime component (τ) should be solely triplet state emission because τ in Au_28ii_ decreases from 2.4 to ∼1.6 μs from He to O_2_, which validates its phosphorescence nature.

### Temperature-Dependent PL of the Isomeric Au_28_(SR)_20_ NCs

To gain further insights into the origin of PL, temperature-dependent PL spectra for the isomeric NCs were measured from room temperature (rt, 290 K) down to 80 K. The NCs were dissolved in 2-methyltetrahydrofuran (2-MeTHF) to form clear “glass” at cryogenic temperatures for optical measurements. The NCs showed no noticeable degradation after cryogenic measurements, as evidenced by superimposable UV–vis spectra before/after the tests (Figure S2).

**Figure 4:**
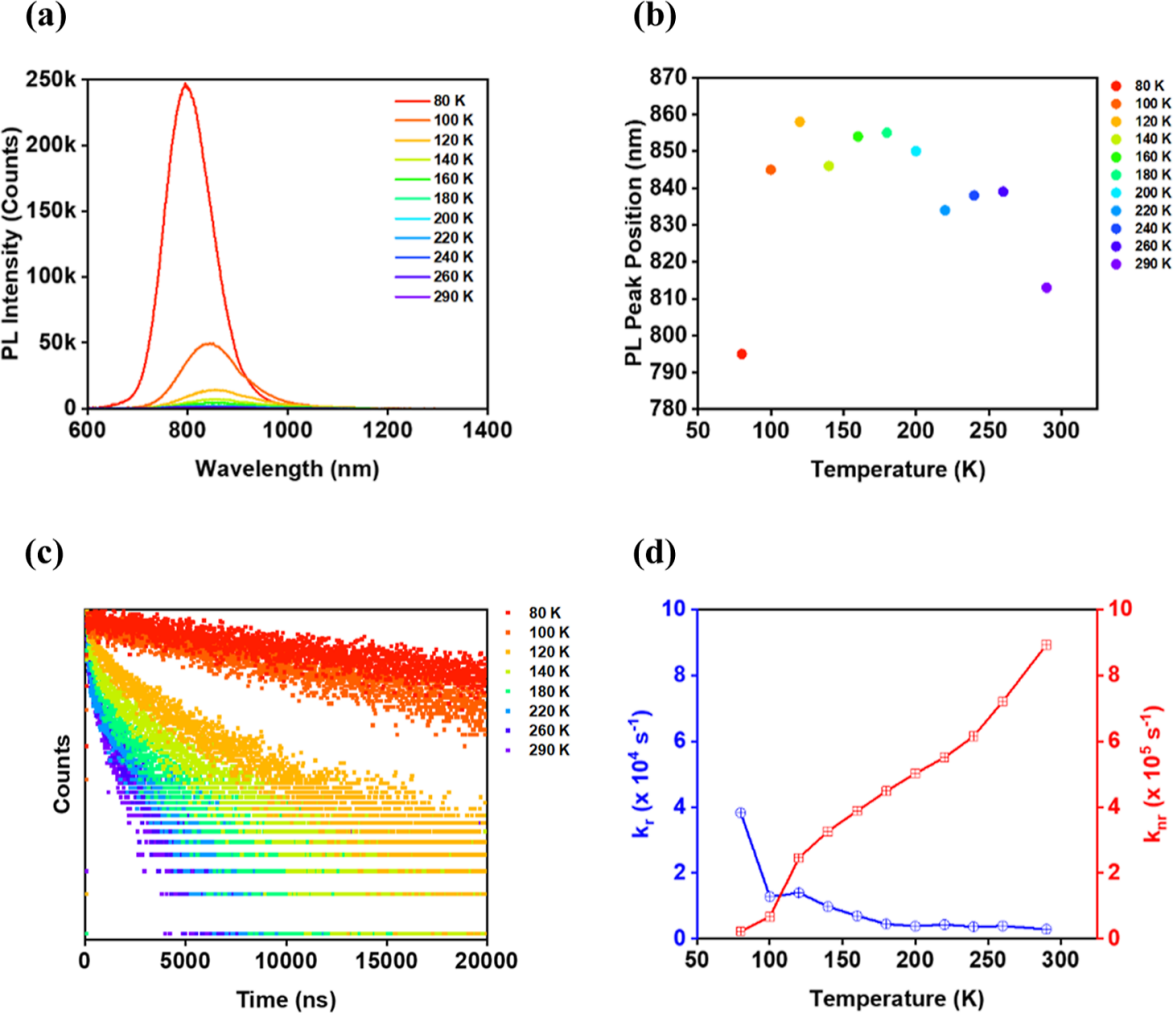
Temperature-dependent data for **Au**_**28i**_**(CHT)**_**20**_. (a) PL spectra in 2-MeTHF of varying temperature from 290 to 80 K. (b) Plot of the PL peak position against temperature. (c) PL decay profiles at selected temperatures. (d) Plot of radiative decay rate constants (depicted in blue) and nonradiative decay rate constants (depicted in red) spanning 80 to 290 K.

With a decrease in temperature, the PL peak of Au_28i_ significantly intensifies and also becomes sharper (Figure 4a). The PL peak position shows initially a general redshift from rt to 120 K, but then a blueshift as the temperature decreases further to 80 K (Figure 4b). This trend indicates the presence of TADF in Au_28i_.^1^ The integrated intensity of the PL peak increased monotonically by 2.4 times from rt to 200 K, and further increased by almost 88.2 times from 200 to 80 K; thus, the overall PLQY almost increased by 212 times from 290 to 80 K, that is, from 0.3 to 63.5%. The PL lifetime becomes much longer at low temperatures (Figure 4c), indicating a significant suppression of the nonradiative relaxation by staple vibrations. Overall, the drastic enhancement of the PLQY at 80 K is contributed by the significant suppression of the staple vibrations along with an increase of the radiative rate at low temperature (Figure 4d).

**Figure 5:**
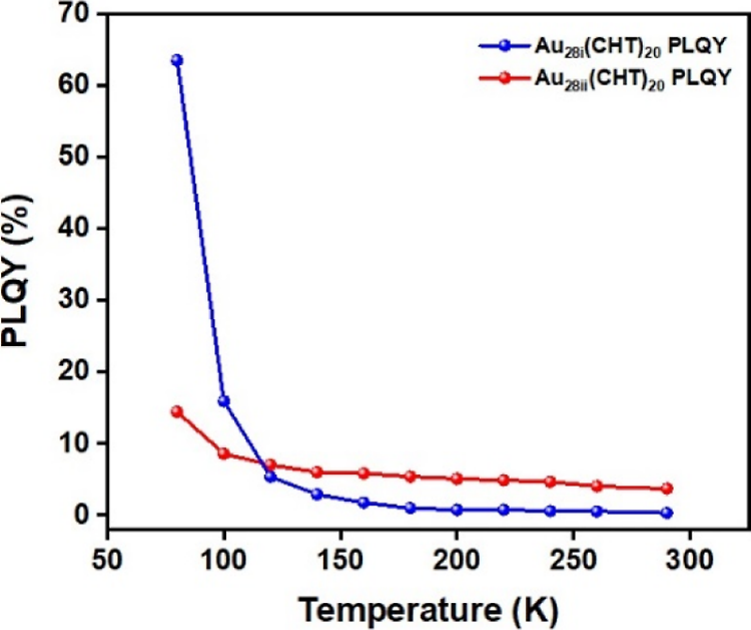
Temperature-dependent PL quantum yields of **Au**_**28i**_**(CHT)**_**20**_ (denoted as blue) vs **Au**_**28ii**_**(CHT)**_**20**_ (denoted as red) in 2-MeTHF solution.

In the case of Au_28ii_ (Figure S3a,b), as the temperature decreased, the peak position remained unchanged from rt to 220 K and then red-shifted from 220 to 120 K, and finally a slight blueshift down to 80 K. The PL lifetime became longer with decreasing temperature (Figure S3c). In this case, as only one emission lifetime is observed throughout the temperature range from 290 to 80 K and the lifetime is of microseconds, we assign this decay pathway as phosphorescence. Unlike Au_28i_, there is no TADF in Au_28ii_ due to the lack of an additional short lifetime component and any variation with temperature in the PLQY as would be expected for TADF. Upon calculating *k*_r_ and *k*_nr_ at different temperatures (Figure S3d), it was observed that in this instance, there is a suppression of the nonradiative pathway without a substantial increase in the radiative pathway. A monotonic increment in PL peak intensity was observed, but only a moderate 3.9-fold enhancement of PLQY was found (Figure S3e). Overall, Au_28ii_ only exhibits a relatively modest enhancement in PLQY (i.e., from 3.7 to 14.5%) compared to the Au_28i_ case (Figure 5).

To obtain further insights into whether or not the 212 times enhancement of the Au_28i_ QY is due to the absorption increase at 80 K, we conducted cryogenic absorption measurements (Figure S4a), which showed only a 17.8% increase in absorbance at 365 nm (the excitation wavelength for PL) from 290 K down to 80 K. Thus, the 212-fold increase is not due to the absorption enhancement but due to the suppression of nonradiative decay by staple vibrations, especially the high frequency vibrations of the four dimeric staples in Au_28i_. The temperature-dependent PL excitation spectra (Figure S4b) of Au_28i_ are essentially unchanged with decreasing temperature, and all show similar spectral profiles, also being similar as that of the absorption spectrum, indicating that the observed PL emission comes from the first excited state (singlet and triplet) over the entire temperature range.

### Insights into the PL Mechanism of the Isomeric Au_28_(SR)_20_ NCs

The above results show that Au_28i_ is less emissive at rt (PLQY 0.3%) but becomes highly emissive (PLQY 63.5%) at 80 K (enhanced by 212 times) and, hence, very sensitive to temperature. In contrast, Au_28ii_ is more emissive at rt (PLQY = 3.7%) but only exhibits a modest enhancement (3.9 times, PLQY = 14.5%) at 80 K, thus being not as sensitive to temperature. To gain further insights into their differences, we further carried out cryogenic absorption measurements on Au_28ii_ for comparison with Au_28i_. The cryo-measurements can probe the vibrational modes that are coupled to the electronic transitions.

In the temperature-dependent absorption spectra of Au_28i_ (Figure S4a), an obvious blueshift (in wavelength) of the HOMO–LUMO transition peak (i.e., *E*_g_) is evident for Au_28i_ as the temperature decreases from rt down to 80 K. In contrast, the corresponding gap value for Au_28ii_ remains nearly constant (Figure S5a). Generally speaking, the renormalization of the *E*_g_ value is a direct reflection of the electron-vibration interaction in the materials. The large blueshift of the gap in Au_28i_ suggests a high-frequency vibrational mode in coupling with the band gap electronic transition, which can be efficiently suppressed as the temperature drops to 80 K. On the other hand, the relatively constant gap value of Au_28ii_ implies that the coupled vibration is of low frequency and cannot be efficiently suppressed at 80 K, unless lower temperatures are applied. These insights explain the observed temperature-dependent PL in which the PLQY of Au_28i_ increases rapidly with the temperature drop and reaches 63.5% at 80 K (i.e., the staple vibration-induced nonradiative decay is largely suppressed), while the PLQY of Au_28ii_ increases much more slowly with the temperature drop because low-frequency vibrations are not effectively suppressed at 80 K unless lower temperatures are applied.

Based on the above discussions, for Au_28i_, the ∼256 ns lifetime at rt under a N_2_ atmosphere (Table S1) should originate from the radiative relaxation of the first singlet excited state (S_1_), while the ∼1.4 μs should be from the radiative relaxation of the first triplet excited state (T_1_), and for Au_28ii_ the sole lifetime component (∼1.9 μs) should be from the radiative relaxation of T_1_. Based on the temperature-dependent PL decay curves in the 290 to 80 K range (Figure 3c for Au_28i_ and Figure S3c for Au_28ii_), the fitting results for both NCs are listed in Table S1. In the case of Au_28i_, as the temperature decreases from rt down to 120 K, the two components become longer and the percentage of τ_2_ increases rapidly, while the percentage of τ_1_ decreases. When the temperature is lower than 120 K, only one component (i.e., τ_2_) remains, whereas the other radiative process (i.e., τ_1_) is suppressed completely; thus, we ascribe the observed τ_1_ to a TADF process. On the other hand, for Au_28ii_, only one component (τ) was found throughout the temperature range from 290 to 80 K and it became longer at lower temperatures.

Thermally activated delayed fluorescence (TADF) necessitates effective ISC (S_1_ to T_1_) and an extremely narrow gap (<0.2 eV) between S_1_ and T_1_. This enables thermal energy to replenish the S_1_ state through an “uphill” transfer of the T_1_ population, a process known as reverse ISC (RISC).^37,38^ The occurrence of TADF in Au_28i_ suggests the proximity of the S_1_ and T_1_ states and efficient population of these states above 120 K. However, below 120 K, the RISC process was suppressed due to insufficient thermal energy, and the emission mainly arises from the triplet state (i.e., phosphorescence) in the Au_28i_. Here, a pertinent question arises: why was TADF not detected in Au_28ii_? To address this and also gain a deeper understanding of the PL mechanism, DFT and time-dependent DFT (TD-DFT) calculations were conducted on these isomeric Au_28_(CHT)_20_ NCs.

### Theoretical Calculations and PL Mechanism of the Isomeric Au_28_(SR)_20_ NCs

In recent research on atomically precise NCs, theoretical calculations have played a critical role in revealing the structure and electronic and dynamic properties of metal nanoclusters.^39−51^ Here, the structures of the ground state (S_0_) and excited states (S_1_, T_1_, and T_2_) of the two isomers of Au_28_ were optimized by DFT and TD-DFT methods. The energy difference between S_1_ and T_1_, Δ*E*_S–T_(S_1_–T_1_), were obtained. It is found that the calculated Δ*E*_S–T_(S_1_–T_1_) value of Au_28i_ (0.126 eV) is smaller than that of Au_28ii_ (0.240 eV); thus, we expect efficient RISC in Au_28i_ but not in Au_28ii_. For both Au_28i_ and Au_28ii_, the main contributions to S_1_ → S_0_ and T_1_ → S_0_ transitions originate from the LUMO → HOMO transition (Table 2). Therefore, we computed the centroid distance between the HOMO and LUMO orbitals (Figure S6). The Au_28i_ shows a larger HOMO–LUMO centroid distance (0.53 Å) than Au_28ii_ (0.15 Å). The latter smaller HOMO–LUMO centroid distance results in a greater electron exchange interaction in Au_28ii_, leading to a larger Δ*E*_S-T_ value, while the larger HOMO–LUMO centroid distance yields a smaller Δ*E*_S-T_ value for Au_28i_.

**Table 2:** Computed Vertical Emission Energy, Emission Wavelength, and Major Orbital Contributions

	transition	emission energy (eV)	wavelength (nm)	major orbital contributions
**Au**_**28i**_	S_1_ → S_0_	1.583	783	L → H (99.08%)
	T_1_ → S_0_	1.487	834	L → H (98.02%)
**Au**_**28ii**_	S_1_ → S_0_	1.678	739	L → H (99.05%)
	T_1_ → S_0_	1.316	942	L → H (98.08%)

In Table 3, the computed spin orbit coupling matrix element (SOCME), the state energy difference Δ*E*, ISC, RISC, IC, and RIC rate constants of Au_28i_ and Au_28ii_ are presented. Due to the significant relativistic effect of Au, the SOCME between S_1_ and T_2_, T_1_ are large for both Au_28i_ and Au_28ii_. Such large SOCME values result in very fast ISC processes for Au_28i_ and Au_28ii_. It is worth noting that S_1_ → S_0_ and T_1_ → S_0_ possess the same orbital contributions (L → H), which results in a much smaller SOCME between S_1_ and T_1_ than that between S_1_ and T_2_; this can be understood from the El-Sayed rule.

**Table 3:** Computed SOCME, Δ*E*, ISC, RISC, IC, and RIC Rate Constants

	transition	SOCME (cm^–1^)	Δ*E* (eV)	rate constant (s^–1^)
**Au**_**28i**_	S_1_ → T_1_ ISC	25.34	0.126	3.53 × 10^10^
	T_1_ → S_1_ RISC	22.88		2.90 × 10^10^
	S_1_ → T_2_ ISC	221.99	0.135	1.74 × 10^12^
	T_2_ → S_1_ RISC	369.10		4.81 × 10^12^
	T_2_ → T_1_ IC		0.261	1.75 × 10^11^
	T_1_ → T_2_ RIC			1.45 × 10^7^
	T_1_ → S_0_ radiation	109.32		4.61 × 10^3^
**Au**_**28ii**_	S_1_ → T_1_ ISC	1.05	0.240	2.14 × 10^4^
	T_1_ → S_1_ RISC	71.15		9.87 × 10^3^
	S_1_ → T_2_ ISC	166.26	0.051	1.92 × 10^13^
	T_2_ → S_1_ RISC	37.14		1.92 × 10^11^
	T_2_ → T_1_ IC		0.189	1.64 × 10^13^
	T_1_ → T_2_ RIC			6.38 × 10^8^
	T_1_ → S_0_ radiation	187.71		3.83 × 10^4^

**Figure 6:**
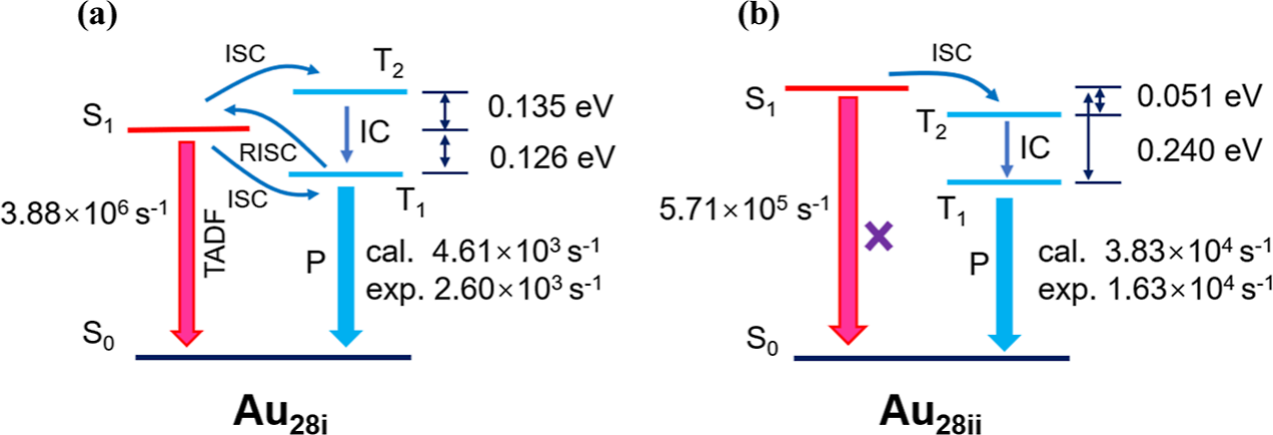
Simulated mechanism of photoluminescence for (a) **Au**_**28i**_ and (b) **Au**_**28ii**_.

For Au_28i_, the computed S_1_ → T_1_ and S_1_ → T_2_ ISC rate constants  and  are 3.53 × 10^10^ s^–1^ and 1.74 × 10^12^ s^–1^, respectively. The larger ISC rate constant  can be attributed to larger SOCME of S_1_ and T_2_ (221.99 cm^–1^), which indicates that the S_1_ → T_2_ ISC process is very favorable. After the ISC from S_1_ and T_2_, an internal conversion (IC) process is expected from T_2_ → T_1_. Here, the computed T_2_ → T_1_ IC rate constant  is 1.75 × 10^11^ s^–1^. Starting from T_1_, the direct emission from T_1_ → S_0_, the RISC from T_1_ → S_1_, and the reverse internal conversion (RIC) processes are further studied. In Table 3, we display the computed rate constants of these processes. Due to the relatively large energy difference between T_2_ and T_1_ (0.261 eV), the T_1_ → T_2_ RIC rate constant  (1.45 × 10^7^ s^–1^) is much lower than  (1.75 × 10^11^ s^–1^). The computed  of Au_28i_ (2.9 × 10^10^ s^–1^) is very close to the  (3.53 × 10^10^ s^–1^) and far greater than the phosphorescence radiative rate constant *k*_P_ (T_1_ → S_0_, 4.61 × 10^3^ s^–1^). Taking these computed rate constants together, an indirect conversion path from S_1_ to T_1_ by way of T_2_, namely, S_1_ → T_2_ → T_1_ process is suggested (Figure 6a). It is worth noting that the contribution of higher triplet states to the ISC process has also been reported in other NCs.^52−54^

For Au_28ii_, because of relatively small SOCME (1.05 cm^–1^) and relatively large Δ*E*_S–T_(S_1_–T_1_), the S_1_ → T_1_ ISC rate constant  is only 2.14 × 10^4^ s^–1^, which is lower than the fluorescence radiative rate constant *k*_F_ (5.71 × 10^5^ s^–1^), indicating very low possibility of S_1_ reaching T_1_ through the ISC process. However, a very small Δ*E*_S–T_ between S_1_ and T_2_ (0.051 eV) induces a large S_1_ → T_2_ ISC rate constant  (1.92 × 10^13^ s^–1^), which is beneficial to the S_1_ → T_2_ ISC process (Figure 6b). Subsequently, similar to Au_28i_, the larger  and  (1.64 × 10^13^ s^–1^) open an effective path for S_1_ to indirectly convert into T_1_ through T_2_. Finally, due to the large Δ*E*_S–T_(S_1_–T_1_), the T_1_ → S_1_ RISC rate constant  (9.87 × 10^3^ s^–1^) is less than  (2.14 × 10^4^ s^–1^) and phosphorescence radiative rate constant *k*_P_ (T_1_ → S_0_, 3.83 × 10^4^ s^–1^). This situation will make the T_1_ → S_1_ RISC process unable to compete with the S_1_ → T_1_ ISC and the T_1_ → S_0_ phosphorescence radiative process. A direct phosphorescence radiation process eventually resulted in the Au_28ii_.

**Figure 7:**
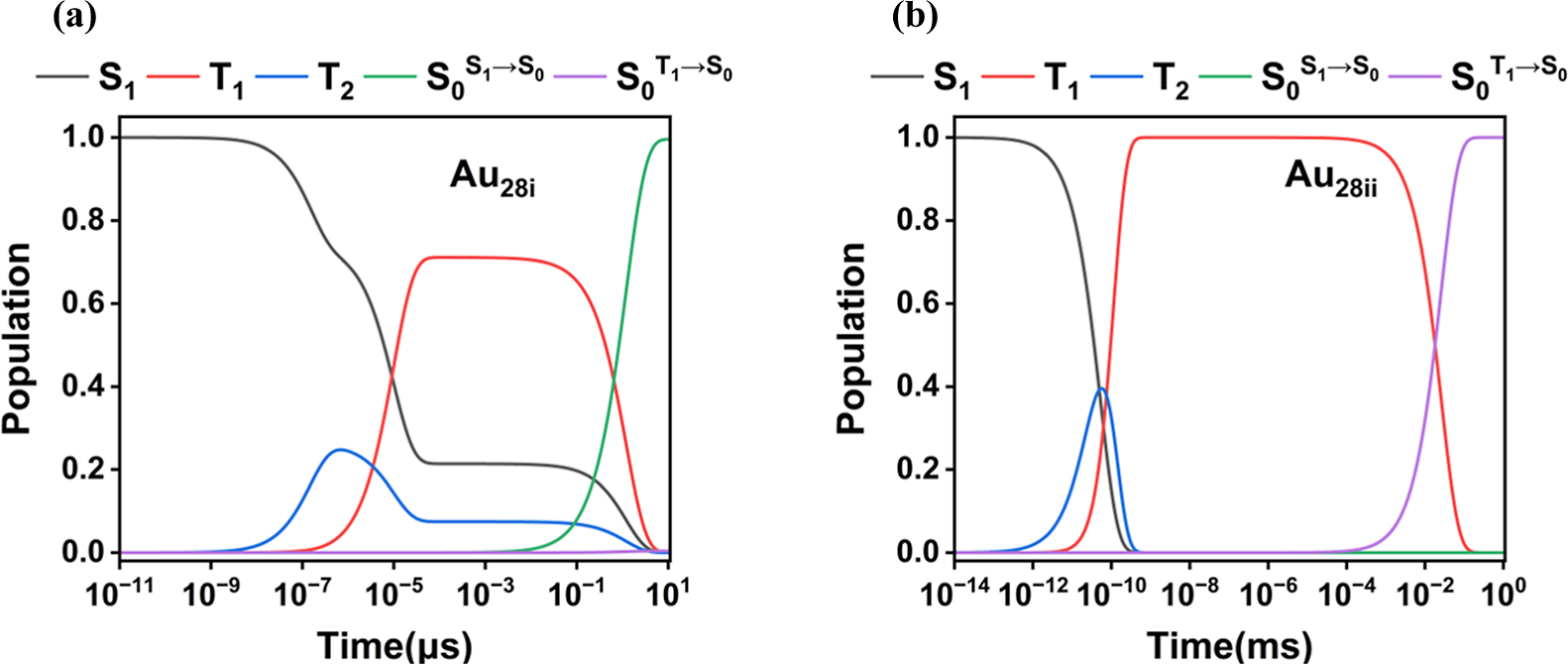
Dynamic evolution diagram for (a) **Au**_**28i**_ and (b) **Au**_**28ii**_.

Taking the above discussions together, it is found that the T_2_ state plays a significant role in the photophysical processes of Au_28i_ and Au_28ii_. The fast S_1_ → T_2_ ISC provides an indirect pathway from S_1_ to T_1_, including the ISC from S_1_ to T_2_ first and then from T_2_ to T_1_ via an IC process. However, due to the different Δ*E*_S–T_(S_1_–T_1_) values in Au_28i_ and Au_28ii_, the RISC rate constants of T_1_ to S_1_ of the two NCs differ greatly, which leads to different luminescence mechanisms (Figure 6). Based on the computed rate constants, we plotted the dynamic evolution diagram for Au_28i_ and Au_28ii_ (Figure 7). The dynamic evolution diagram can better describe the photophysical processes of Au_28i_ and Au_28ii_ and explain the differences in their photoluminescence types. For Au_28i_ and Au_28ii_, since the  is larger than , T_2_ population accumulates rapidly with S_1_ population decreasing rapidly; then, the T_2_ population decreases gradually with the increase in T_1_ population. Finally, the T_1_ population gradually decreases to 0, with the S_0_ population increasing to 1. For Au_28i_, the increase in the S_0_ population is primarily attributed to the S_1_ → S_0_ radiative transition, indicating a fluorescence process, specifically TADF emission (T_1_ → S_1_ → S_0_). Additionally, the ratio of the S_0_ population arising from S_1_ radiative transition compared to that from T_1_ transition is 2500:1, suggesting the coexistence of T_1_ → S_0_ phosphorescence. For Au_28ii_, the increase in the S_0_ population is primarily due to T_1_ → S_0_ radiative transition, with the ratio of the S_0_ population from S_1_ radiative transition to that from T_1_ transition being 6:1,000,000.

To understand the significant difference in phosphorescence efficiency between Au_28i_ and Au_28ii_ at low temperatures, we calculated the Huang–Rhys factor (i.e., electron-vibration coupling strength), the reorganization energy, and the Dushinsky matrix for the T_1_ → S_0_ ISC process. Unlike Au_28i_, the case of Au_28ii_ exhibits significant reorganization energy contributions for S–Au and S–C stretching vibrations (Figure S7). As the temperature decreases, high-frequency vibration modes are suppressed, while in the low-frequency region the Dushinsky rotational effect of Au_28ii_ is larger than that of Au_28i_ (Figure S8). Consequently, at low temperatures, the nonradiative processes of Au_28ii_ cannot be effectively suppressed, resulting in a significant difference in phosphorescence efficiency between Au_28i_ and Au_28ii_.

## Conclusions

In summary, this work reports the structural isomeric effects on the near-infrared PLQY in the two isomeric Au_28_(CHT)_20_ nanoclusters, and their PL mechanisms are established through a combined analysis by experiment and DFT/TD-DFT simulations. The Au_28i_ exhibits a low PLQY (0.3%), whereas its structural isomer Au_28ii_ exhibits a relatively high PLQY (3.7%) at room temperature under ambient conditions in solution, but at cryogenic temperatures, the vibration-induced nonradiative relaxation is more effectively suppressed in Au_28i_ than in Au_28ii_, leading to a switch of the order of PL intensity, that is, a ∼212-fold enhancement for Au_28i_ (PLQY: 63.5%) at 80 K versus merely a 3.9-fold enhancement in Au_28ii_ (PLQY: 14.5%) at 80 K. Temperature-dependent PL measurements along with theoretical calculations reveal both TADF and phosphorescence emission in Au_28i_ but sole phosphorescence in Au_28ii_ due to their different S_1_–T_1_ gap energies. In both NCs, theoretical simulations indicate a very efficient indirect S_1_ → T_2_ → T_1_ conversion process, but efficient RISC occurs only in Au_28i_ due to its favorable S_1_–T_1_ gap (<0.2 eV), hence, concurrent TADF and phosphorescence in Au_28i_, in contrast with the sole phosphorescence in Au_28ii_ due to suppressed RISC because of a larger S_1_–T_1_ gap. Overall, this work presents a paradigm for investigating the complex PL mechanism via a combined experiment-theory approach, and the obtained mechanisms and isomeric effect in enhancing NIR-luminescence will promote the design of NIR emitters and the development of their applications in sensing, bioimaging, photonics, and other fields.

## Methods/Experimental Section

The synthesis and isolation of isomeric Au_28i_ and Au_28ii_ nanoclusters followed a literature protocol.^26^ Spectroscopic characterization includes optical absorption, steady-state, and time-resolved photoluminescence, as well as cryogenic spectroscopy. DFT simulations were carried out. Details are provided in the Supporting Information.

## Supporting Information Available

Details of the synthesis, spectroscopic measurements, and theoretical simulations (PDF)

## References

[ref1] Liu Z.; Luo L.; Jin R. Visible to NIR-II Photoluminescence of Atomically Precise Gold Nanoclusters. Adv. Mater. 2023, 36, 2309073 10.1002/adma.202309073. 37922431

[ref2] Ziefuss A. R.; Steenbock T.; Benner D.; Plech A.; Göttlicher J.; Teubner M.; Grimm-Lebsanft B.; Rehbock C.; Comby-Zerbino C.; Antoine R.; Amans D.; Chakraborty I.; Bester G.; Nachev M.; Sures B.; Rübhausen M.; Parak W. J.; Barcikowski S. Photoluminescence of Fully Inorganic Colloidal Gold Nanocluster and Their Manipulation Using Surface Charge Effects. Adv. Mater. 2021, 33, 2101549 10.1002/adma.202101549. PMC11469328 34165866

[ref3] Aminfar P.; Ferguson T.; Steele E.; MacNeil E. M.; Matus M. F.; Malola S.; Häkkinen H.; Duchesne P. N.; Loock H.-P.; Stamplecoskie K. G. Accelerated Size-Focusing Light Activated Synthesis of Atomically Precise Fluorescent Au_22_(Lys–Cys–Lys)_16_ Clusters. Nanoscale 2024, 16, 205–211. 10.1039/D3NR04793H. 38051125

[ref4] Crawford S. E.; Hartmann M. J.; Millstone J. E. Surface Chemistry-Mediated Near-Infrared Emission of Small Coinage Metal Nanoparticles. Acc. Chem. Res. 2019, 52, 695–703. 10.1021/acs.accounts.8b00573. 30742413

[ref5] Zhao J.; Zhong D.; Zhou S. NIR-I-to-NIR-II Fluorescent Nanomaterials for Biomedical Imaging and Cancer Therapy. J. Mater. Chem. B 2018, 6, 349–365. 10.1039/C7TB02573D. 32254515

[ref6] Luo X.; Kong J.; Xiao H.; Sang D.; He K.; Zhou M.; Liu J. Noncovalent Interaction Guided Precise Photoluminescence Regulation of Gold Nanoclusters in Both Isolate Species and Aggregate States. Angew. Chem., Int. Ed. 2024, 63, e202404129 10.1002/anie.202404129. 38651974

[ref7] Liu J.; Yu M.; Zhou C.; Yang S.; Ning X.; Zheng J. Passive Tumor Targeting of Renal-Clearable Luminescent Gold Nanoparticles: Long Tumor Retention and Fast Normal Tissue Clearance. J. Am. Chem. Soc. 2013, 135, 4978–4981. 10.1021/ja401612x. 23506476 PMC4127425

[ref8] Luo Z.; Zheng K.; Xie J. Engineering Ultrasmall Water-Soluble Gold and Silver Nanoclusters for Biomedical Applications. Chem. Commun. 2014, 50, 5143–5155. 10.1039/C3CC47512C. 24266029

[ref9] Yang G.; Pan X.; Feng W.; Yao Q.; Jiang F.; Du F.; Zhou X.; Xie J.; Yuan X. Engineering Au _44_ Nanoclusters for NIR-II Luminescence Imaging-Guided Photoactivatable Cancer Immunotherapy. ACS Nano 2023, 17, 15605–15614. 10.1021/acsnano.3c02370. 37503901

[ref10] Luo L.; Liu Z.; Kong J.; Gianopoulos C. G.; Coburn I.; Kirschbaum K.; Zhou M.; Jin R. Three-Atom-Wide Gold Quantum Rods with Periodic Elongation and Strongly Polarized Excitons. Proc. Natl. Acad. Sci. U.S.A. 2024, 121, e2318537121 10.1073/pnas.2318537121. 38412123 PMC10927531

[ref11] Xu M.-M.; Jia T.-T.; Li B.; Ma W.; Chen X.; Zhao X.; Zang S.-Q. Tuning the Properties of Atomically Precise Gold Nanoclusters for Biolabeling and Drug Delivery. Chem. Commun. 2020, 56, 8766–8769. 10.1039/D0CC03498C. 32613976

[ref12] Liu Z.; Zhou M.; Luo L.; Wang Y.; Kahng E.; Jin R. Elucidating the Near-Infrared Photoluminescence Mechanism of Homometal and Doped M_25_ (SR)_18_ Nanoclusters. J. Am. Chem. Soc. 2023, 145, 19969–19981. 10.1021/jacs.3c06543. 37642696 PMC10510323

[ref13] Zhou M.; Higaki T.; Li Y.; Zeng C.; Li Q.; Sfeir M. Y.; Jin R. Three-Stage Evolution from Nonscalable to Scalable Optical Properties of Thiolate-Protected Gold Nanoclusters. J. Am. Chem. Soc. 2019, 141, 19754–19764. 10.1021/jacs.9b09066. 31809035

[ref14] Lin H.; Song X.; Chai O. J. H.; Yao Q.; Yang H.; Xie J. Photoluminescent Characterization of Metal Nanoclusters: Basic Parameters, Methods, and Applications. Adv. Mater. 2024, 36, 2401002 10.1002/adma.202401002. 38521974

[ref15] Liu Z.; Li Y.; Kahng E.; Xue S.; Du X.; Li S.; Jin R. Tailoring the Electron–Phonon Interaction in Au_25_(SR)_18_ Nanoclusters via Ligand Engineering and Insight into Luminescence. ACS Nano 2022, 16, 18448–18458. 10.1021/acsnano.2c06586. 36252530

[ref16] Wang Y.; Liu Z.; Mazumder A.; Gianopoulos C. G.; Kirschbaum K.; Peteanu L. A.; Jin R. Tailoring Carbon Tails of Ligands on Au _52_ (SR) _32_ Nanoclusters Enhances the Near-Infrared Photoluminescence Quantum Yield from 3.8 to 18.3. J. Am. Chem. Soc. 2023, 145, 26328–26338. 10.1021/jacs.3c09846. 37982713 PMC10704554

[ref17] Zhang B.; Chen J.; Cao Y.; Chai O. J. H.; Xie J. Ligand Design in Ligand-Protected Gold Nanoclusters. Small 2021, 17, 2004381 10.1002/smll.202004381. 33511773

[ref18] Jin S.; Liu W.; Hu D.; Zou X.; Kang X.; Du W.; Chen S.; Wei S.; Wang S.; Zhu M. Aggregation-Induced Emission (AIE) in Ag–Au Bimetallic Nanocluster. Chem.—Eur. J. 2018, 24, 3712–3715. 10.1002/chem.201800189. 29392775

[ref19] Mitsui M.; Arima D.; Kobayashi Y.; Lee E.; Niihori Y. On the Origin of Photoluminescence Enhancement in Biicosahedral Ag_x_Au_25–x_ Nanoclusters (*x* = 0–13) and Their Application to Triplet–Triplet Annihilation Photon Upconversion. Adv. Opt. Mater. 2022, 10, 2200864 10.1002/adom.202200864.

[ref20] Takano S.; Hirai H.; Nakashima T.; Iwasa T.; Taketsugu T.; Tsukuda T. Photoluminescence of Doped Superatoms M@Au_12_ (M = Ru, Rh, Ir) Homoleptically Capped by (Ph_2_)PCH_2_P(Ph_2_): Efficient Room-Temperature Phosphorescence from Ru@Au _12_. J. Am. Chem. Soc. 2021, 143, 10560–10564. 10.1021/jacs.1c05019. 34232036

[ref21] Chakraborty S.; Bain D.; Maity S.; Kolay S.; Patra A. Controlling Aggregation-Induced Emission in Bimetallic Gold–Copper Nanoclusters via Surface Motif Engineering. J. Phys. Chem. C 2022, 126, 2896–2904. 10.1021/acs.jpcc.1c10237.

[ref22] Luo L.; Liu Z.; Du X.; Jin R. Near-Infrared Dual Emission from the Au_42_(SR)_32_ Nanocluster and Tailoring of Intersystem Crossing. J. Am. Chem. Soc. 2022, 144, 19243–19247. 10.1021/jacs.2c09107. 36239690

[ref23] Hirai H.; Takano S.; Nakashima T.; Iwasa T.; Taketsugu T.; Tsukuda T. Doping-Mediated Energy-Level Engineering of M@Au_12_ Superatoms (M = Pd, Pt, Rh, Ir) for Efficient Photoluminescence and Photocatalysis. Angew. Chem., Int. Ed. 2022, 61, e202207290 10.1002/anie.202207290. 35608869

[ref24] Guo Y.; Ma Z.; Niu X.; Zhang W.; Tao M.; Guo Q.; Wang Z.; Xia A. Bridge-Mediated Charge Separation in Isomeric *N*-Annulated Perylene Diimide Dimers. J. Am. Chem. Soc. 2019, 141, 12789–12796. 10.1021/jacs.9b05723. 31334641

[ref25] Yousefalizadeh G.; Stamplecoskie K. G. Photophysics of Ag and Au Alloys of M_25_(SR)_18_ Clusters. J. Chem. Phys. 2021, 155, 134301 10.1063/5.0059624. 34624992

[ref26] Xia N.; Yuan J.; Liao L.; Zhang W.; Li J.; Deng H.; Yang J.; Wu Z. Structural Oscillation Revealed in Gold Nanoparticles. J. Am. Chem. Soc. 2020, 142, 12140–12145. 10.1021/jacs.0c02117. 32517466

[ref27] Li J.; Wang P.; Pei Y. Ligand Shell Isomerization Induces Different Fluorescence Origins of Two Au_28_ Nanoclusters. J. Phys. Chem. Lett. 2022, 13, 3718–3725. 10.1021/acs.jpclett.2c00539. 35442683

[ref28] Xu W. W.; Zeng X. C.; Gao Y. The Structural Isomerism in Gold Nanoclusters. Nanoscale 2018, 10, 9476–9483. 10.1039/C8NR02284D. 29637968

[ref29] Kang X.; Zhu M. Structural Isomerism in Atomically Precise Nanoclusters. Chem. Mater. 2021, 33, 39–62. 10.1021/acs.chemmater.0c03979.

[ref30] Chen Y.; Liu C.; Tang Q.; Zeng C.; Higaki T.; Das A.; Jiang D.; Rosi N. L.; Jin R. Isomerism in Au _28_ (SR) _20_ Nanocluster and Stable Structures. J. Am. Chem. Soc. 2016, 138, 1482–1485. 10.1021/jacs.5b12094. 26817394

[ref31] Chen Y.; Zhou M.; Li Q.; Gronlund H.; Jin R. Isomerization-Induced Enhancement of Luminescence in Au_28_(SR)_20_ Nanoclusters. Chem. Sci. 2020, 11, 8176–8183. 10.1039/D0SC01270J. 34123088 PMC8163317

[ref32] Li Q.; Zeman C. J.; Ma Z.; Schatz G. C.; Gu X. W. Bright NIR-II Photoluminescence in Rod-Shaped Icosahedral Gold Nanoclusters. Small 2021, 17, 2007992 10.1002/smll.202007992. 33620777

[ref33] Han X.; Luan X.; Su H.; Li J.; Yuan S.; Lei Z.; Pei Y.; Wang Q. Structure Determination of Alkynyl-Protected Gold Nanocluster Au_22_(^t^BuC≡C)_18_ and Its Thermochromic Luminescence. Angew. Chem., Int. Ed. 2020, 59, 2309–2312. 10.1002/anie.201912984. 31769148

[ref34] Deaton J. C.; Switalski S. C.; Kondakov D. Y.; Young R. H.; Pawlik T. D.; Giesen D. J.; Harkins S. B.; Miller A. J. M.; Mickenberg S. F.; Peters J. C. E-Type Delayed Fluorescence of a Phosphine-Supported Cu_2_(μ-NAr_2_)_2_ Diamond Core: Harvesting Singlet and Triplet Excitons in OLEDs. J. Am. Chem. Soc. 2010, 132, 9499–9508. 10.1021/ja1004575. 20557045

[ref35] Luo L.; Liu Z.; Du X.; Jin R. Photoluminescence of the Au_38_(SR)_26_ Nanocluster Comprises Three Radiative Processes. Commun. Chem. 2023, 6, 22 10.1038/s42004-023-00819-3. 36732442 PMC9894927

[ref36] Zhang L. L. M.; Zhou G.; Zhou G.; Lee H. K.; Zhao N.; Prezhdo O. V.; Mak T. C. W. Core-Dependent Properties of Copper Nanoclusters: Valence-Pure Nanoclusters as NIR TADF Emitters and Mixed-Valence Ones as Semiconductors. Chem. Sci. 2019, 10, 10122–10128. 10.1039/C9SC03455B. 32055367 PMC7003970

[ref37] Yuan Z.; Wang Z.; Han B.; Zhang C.; Zhang S.; Zhu Z.; Yu J.; Li T.; Li Y.; Tung C.; Sun D. Ag_22_ Nanoclusters with Thermally Activated Delayed Fluorescence Protected by Ag/Cyanurate/Phosphine Metallamacrocyclic Monolayers through In-Situ Ligand Transesterification. Angew. Chem., Int. Ed. 2022, 61, e202211628 10.1002/anie.202211628. 36104622

[ref38] Sevilla R. C.; Soebroto R. J.; Kurniawan I. S.; Chen P. W.; Chang S. H.; Shen J. L.; Chou W. C.; Yeh J. M.; Huang H. Y.; Yuan C. T. Self-Trapped, Thermally Equilibrated Delayed Fluorescence Enables Low-Reabsorption Luminescent Solar Concentrators Based on Gold-Doped Silver Nanoclusters. ACS Appl. Mater. Interfaces 2023, 15, 53136–53145. 10.1021/acsami.3c13710. 37922121

[ref39] Li Y.; Song Y.; Zhang X.; Liu T.; Xu T.; Wang H.; Jiang D. E.; Jin R. Atomically Precise Au_42_ Nanorods with Longitudinal Excitons for an Intense Photothermal Effect. J. Am. Chem. Soc. 2022, 144, 12381–12389. 10.1021/jacs.2c03948. 35767839

[ref40] Liu H.; Wang P.; Pei Y. Mechanism Insight into Metal Exchange between Au_25_(SR)_18_^–^/Ag_25_(SR)_18_^–^ Clusters and Metal Ions from Ab Initio Molecular Dynamics Simulations. Inorg. Chem. 2024, 63, 8625–8635. 10.1021/acs.inorgchem.4c00010. 38684116

[ref41] Wang Y.; Gianopoulos C. G.; Liu Z.; Kirschbaum K.; Alfonso D.; Kauffman D. R.; Jin R. Au_36_(SR)_22_ Nanocluster and a Periodic Pattern from Six to Fourteen Free Electrons in Core Size Evolution. JACS Au 2024, 4, 1928–1934. 10.1021/jacsau.4c00152. 38818069 PMC11134389

[ref42] Zeng Y.; Havenridge S.; Gharib M.; Baksi A.; Weerawardene K. L. D. M.; Ziefuß A. R.; Strelow C.; Rehbock C.; Mews A.; Barcikowski S.; Kappes M. M.; Parak W. J.; Aikens C. M.; Chakraborty I. Impact of Ligands on Structural and Optical Properties of Ag_29_ Nanoclusters. J. Am. Chem. Soc. 2021, 143, 9405–9414. 10.1021/jacs.1c01799. 34138547

[ref43] Xie X. Y.; Cheng K. Q.; Chen W. K.; Li W.; Li Q.; Han J.; Fang W. H.; Cui G. Near-Infrared Dual-Emission of a Thiolate-Protected Au_42_ Nanocluster: Excited States, Nonradiative Rates, and Mechanism. J. Phys. Chem. Lett. 2023, 14, 10025–10031. 10.1021/acs.jpclett.3c02683. 37906639

[ref44] Schaefer B.; Pal R.; Khetrapal N. S.; Amsler M.; Sadeghi A.; Blum V.; Zeng X. C.; Goedecker S.; Wang L. S. Isomerism and Structural Fluxionality in the Au_26_ and Au_26_^–^ Nanoclusters. ACS Nano 2014, 8, 7413–7422. 10.1021/nn502641q. 24960331

[ref45] Patty J. B.; Havenridge S.; Tietje-Mckinney D.; Siegler M. A.; Singh K. K.; Hajy Hosseini R.; Ghabin M.; Aikens C. M.; Das A. Crystal Structure and Optical Properties of a Chiral Mixed Thiolate/Stibine-Protected Au_18_ Cluster. J. Am. Chem. Soc. 2022, 144, 478–484. 10.1021/jacs.1c10778. 34957826

[ref46] Wan X.; Xu W. W.; Yuan S.; Gao Y.; Zeng X.; Wang Q. A Near-Infrared-Emissive Alkynyl-Protected Au_24_ Nanocluster. Angew. Chem. 2015, 127, 9819–9822. 10.1002/ange.201503893. 26119538

[ref47] Pei Y.; Gao Y.; Zeng X. C. Structural Prediction of Thiolate-Protected Au38: A Face-Fused Bi-Icosahedral Au Core. J. Am. Chem. Soc. 2008, 130, 7830–7832. 10.1021/ja802975b. 18517203

[ref48] AbdulHalim L. G.; Bootharaju M. S.; Tang Q.; Del Gobbo S.; AbdulHalim R. G.; Eddaoudi M.; Jiang D. E.; Bakr O. M. Ag_29_(BDT)_12_(TPP)_4_: A Tetravalent Nanocluster. J. Am. Chem. Soc. 2015, 137, 11970–11975. 10.1021/jacs.5b04547. 26104755

[ref49] Zhu M.; Aikens C. M.; Hollander F. J.; Schatz G. C.; Jin R. Correlating the Crystal Structure of A Thiol-Protected Au_25_ Cluster and Optical Properties. J. Am. Chem. Soc. 2008, 130, 5883–5885. 10.1021/ja801173r. 18407639

[ref50] Pihlajamäki A.; Hämäläinen J.; Linja J.; Nieminen P.; Malola S.; Kärkkäinen T.; Häkkinen H.; Häkkinen H. Monte Carlo Simulations of Au_38_(SCH_3_)_24_ Nanocluster Using Distance-Based Machine Learning Methods. J. Phys. Chem. A 2020, 124, 4827–4836. 10.1021/acs.jpca.0c01512. 32412747

[ref51] Li Q.; Mosquera M. A.; Jones L. O.; Parakh A.; Chai J.; Jin R.; Schatz G. C.; Gu X. W. Pressure-Induced Optical Transitions in Metal Nanoclusters. ACS Nano 2020, 14, 11888–11896. 10.1021/acsnano.0c04813. 32790326

[ref52] Zhu C.; Xin J.; Li J.; Li H.; Kang X.; Pei Y.; Zhu M. Fluorescence or Phosphorescence? The Metallic Composition of the Nanocluster Kernel Does Matter. Angew. Chem., Int. Ed. 2022, 61, e202205947 10.1002/anie.202205947. 35596616

[ref53] Yoshida K.; Arima D.; Mitsui M. Dissecting Triplet-State Properties and Intersystem Crossing Mechanism of the Ligand-Protected Au_13_ Superatom. J. Phys. Chem. Lett. 2023, 14, 10967–10973. 10.1021/acs.jpclett.3c02977. 38038710

[ref54] Mitsui M.; Uchida A. Triplet Properties and Intersystem Crossing Mechanism of PtAg_28_ Nanocluster Sensitizers Achieving Low Threshold and Efficient Photon Upconversion. Nanoscale 2024, 16, 3053–3060. 10.1039/D3NR05992H. 38240331

