# Supplementary material for: Isomeric Effects of Au28(S-c-C6H11)20 Nanoclusters on Photoluminescence: Roles of Electron-Vibration Coupling and Higher Triplet State
Source: ACS Nano. 2024 Aug 2;18(32):21534–43. doi: 10.1021/acsnano.4c06702 (PMC11328167; doi:10.1021/acsnano.4c06702)
Supplement: Supplementary file 1 — nn4c06702_si_001.pdf [file nn4c06702_si_001.pdf]

Supporting Information:

Isomeric Effects of Au_{28}(S-*c*-C_6H_{11})$_{20}$ Nanoclusters on Photoluminescence: Roles of Electron-Vibration Coupling and Higher Triplet State

Abhrojyoti Mazumder,[1] Kang Li,[2] Zhongyu Liu,[1] Yitong Wang,[1] Yong Pei,[2]* Linda A. Peteanu,[1]* Rongchao Jin[1]*

[1] Department of Chemistry, Carnegie Mellon University, Pittsburgh PA 15213 USA

[2] Department of Chemistry, Key Laboratory of Environmentally Friendly Chemistry and Applications of MOE, Xiangtan University, Xiangtan, Hunan 411105 China

*Corresponding authors: ypnku78@gmail.com, peteanu@andrew.cmu.edu, rongchao@andrew.cmu.edu

Experimental:

1. Materials and Reagents

Tetrachloroauric (III) acid ($HAuCl_4 \cdot 3H_2O$, 99.999% metal basis, Aldrich), tetraoctylammonium bromide (TOAB, ≥98%, Fluka), cyclohexanethiol ($C_6H_{11}SH$, 98%, Aldrich), sodium borohydride ($NaBH_4$, Aldrich). Solvents: methanol (HPLC grade, ≥99.9%, Aldrich), dichloromethane (DCM, ACS reagent, ≥99.5%, Aldrich), hexane (ACS grade, ≥99.9%, Aldrich), 2-methyltetrahydrofuran (anhydrous, inhibitor-free, ≥99%, Aldrich), chloroform-d (100%, 99.96 atom % D, contains 0.03% (v/v) TMS). All chemicals were used as received without further purification. Nanopure water was prepared using a Barnstead NANOpure Diamond system. Thin-layer chromatography (TLC) plates (silica gel, 250 μm) were obtained from iChromatography.

2. Synthesis of $Au_{28i}(CHT)_{20}$ and $Au_{28ii}(CHT)_{20}$ Nanoclusters

The isomeric $Au_{28}(CHT)_{20}$ nanoclusters were synthesized using a previously reported method with a slight modification.[s1] Briefly, the nanocluster $[Au_{23}(SC_6H_{11})_{16}]^-$ TOA^+ (TOA^+ : tetraoctylammonium) was initially synthesized following a previously reported method.[s2] Subsequently, ~12 mg of the synthesized pure Au_{23} was dissolved in 2 ml of dichloromethane (DCM) in a 50 ml round-bottom flask equipped with a magnetic stir bar. A freshly prepared DCM solution (0.5 ml) containing 2 equivalents of the Au-CHT complex was added to the Au_{23} solution while vigorously stirring at ~500 rpm, and the mixture was stirred for 10 hours at room temperature. The resulting solution was then centrifuged, and the supernatant was collected. TLC was performed using a developing solvent mixture of dichloromethane:hexane = 1:3 (v/v) to separate $Au_{28i}(CHT)_{20}$ and $Au_{28ii}(CHT)_{20}$.

3. Steady-State UV-Vis-NIR Measurements

The UV–vis–NIR spectra for all the Au nanoclusters were obtained using a UV-3600 Plus spectrophotometer (Shimadzu) with a wavelength range of 185-3300 nm. Cryogenic absorption measurements were conducted using a home-assembled system, which included the UV-3600 Plus spectrophotometer, a vacuum pump, an Optistat CF2 cryostat (Oxford Instruments), and a temperature controller.

4. Steady-State and Time-Resolved Photoluminescence and Cryogenic Measurements

Steady-state photoluminescence spectra were recorded using an FLS-1000 spectrofluorometer (Edinburgh). Photoluminescence lifetimes were determined by time-correlated single photon counting (TCSPC) using the same instrument. Near-infrared photoluminescence was captured employing a wide-range InGaAs-based PMT-1700 detector (550-1650 nm) cooled to -80 ℃ with liquid nitrogen. For cryogenic photoluminescence measurements, a home-assembled system was employed, which consists of the FLS-1000 spectrofluorometer, a vacuum pump, an Optistat CF2 cryostat (Oxford Instruments), and a temperature controller.

5. Relative Quantum Yield Determination

The relative quantum yield (Φ_S) of the sample is calculated by using:

$$\boldsymbol{\phi_S = \phi_R \left(\frac{I_S}{I_R}\right)\left(\frac{1-10^{-A_R}}{1-10^{-A_S}}\right)\left(\frac{n_S}{n_R}\right)^2}$$

In this context, $\mathbf{\Phi_R}$ denotes the quantum yield of the reference (standard), **I** represent the integrated photoluminescence (PL) intensity, **A** corresponds to the absorbance of the solution at the excitation wavelength, **n** stands for the refractive index of the solvent, and the subscripts (**S** and **R**) distinguish between the sample and the reference, respectively.

6. Computational Details

Both DFT calculations and TDDFT calculations are done using ORCA5.0.2 software[s3-s4]. The geometric optimization of the ground and excited states of the nanoclusters is carried out using the PBE0[s5] exchange-correlation functional and the def2-SV(P)[s6] basis set. The spin-orbit coupling matrix elements (SOCME) are calculated by using the TDDFT method with PBE0 functional. The DKH-def2-SVP basis set is used for S, C, and H and the SARC-DKH-SVP basis set is used for Au. To improve the computational efficiency, both DFT and TDDFT calculations are performed using the RI (Resolution of Identity) approximation with the auxiliary basis set def2/J[s7]. Nonadiabatic couplings (NAC) between T_2 and T_1 are computed using the PBE0 functional and def2-SVP basis set. All structures are optimized in the gas phase, and the cyclohexyl is simplified to methyl group. The convergence criteria for geometric optimization are set as 1.0×10^{-6} Hartree for energy change and 3.0×10^{-4} Hartree/Å for gradient change, respectively. The geometric optimization of the S_0 and T_1 is performed by using the DFT method, and the structural optimization of S_1 is performed using the TDDFT method. Based on the optimized T_1 and S_0 minimum structure, the frequency calculations for T_1 and S_0 are performed by using Gaussian 16 program[s8] at the same computational level to confirm that optimized structures are minima and related vibration frequencies and modes are also used in subsequent ISC and RISC rate calculations. Then, based on the calculated parameters, including the ΔE_{S-T}, SOCME, NAC and vibrational frequencies, the IC, RIC, ISC and RISC rates are calculated by FCclasses3 program[s9-s11]. Because of the limit of computational cost, the hessian of S_1 and T_2 is replaced by that of T_1.

In terms of dynamic evolution, based on the law of mass action, we establish the dynamic equation of concentration evolution with time for the four states S_1, T_1, T_2 and S_0 according to the conversion relationship (eqs. (1), (2), (3), (4), (5), (6)) between the ground state and the excited state and the rate constant. During the evolution, we select the initial population S_1 as 1. The dynamic evolution process of this work is completed by using our self-designed code.

$$\frac{d}{dt}[S_1] = -\left(k_{S_1 \to T_1} + k_{S_1 \to T_2} + k_f\right)[S_1] + k_{T_1 \to S_1}[T_1] + k_{T_2 \to S_1}[T_2] \tag{1}$$

$$\frac{d}{dt}[T_1] = -\left(k_{T_1 \to S_1} + k_{T_1 \to T_2} + k_{p,T_1}\right)[T_1] + k_{S_1 \to T_1}[S_1] + k_{T_2 \to T_1}[T_2] \tag{2}$$

$$\frac{d}{dt}[T_2] = -\left(k_{T_2 \to S_1} + k_{T_2 \to T_1}\right)[T_1] + k_{T_1 \to T_2}[T_1] + k_{S_1 \to T_2}[S_1] \tag{3}$$

$$\frac{d}{dt}[S_0] = k_f[S_1] + k_{p,T_1}[T_1] \tag{4}$$

$$\frac{d}{dt}[S_0]^{S_1 \to S_0} = k_f[S_1] \tag{5}$$

$$\frac{d}{dt}[S_0]^{T_1 \to S_0} = k_{p,T_1}[T_1] \tag{6}$$

Supporting Figures and Table:

(a) (b)

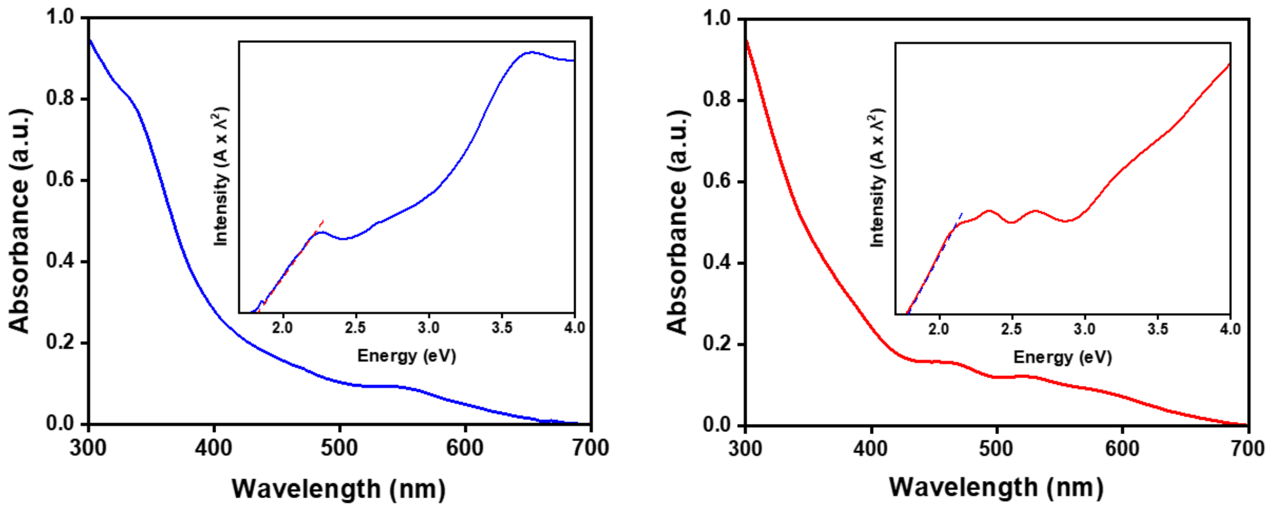

Figure S1. UV-Vis absorption spectrum of (a) **Au_{28i}(CHT)$_{20}$** and (b) **Au_{28ii}(CHT)$_{20}$** in dichloromethane (DCM), respectively. The inset shows the absorption spectrum on the photon energy scale and extrapolation to zero absorbance for E_g determination.

(a) (b)

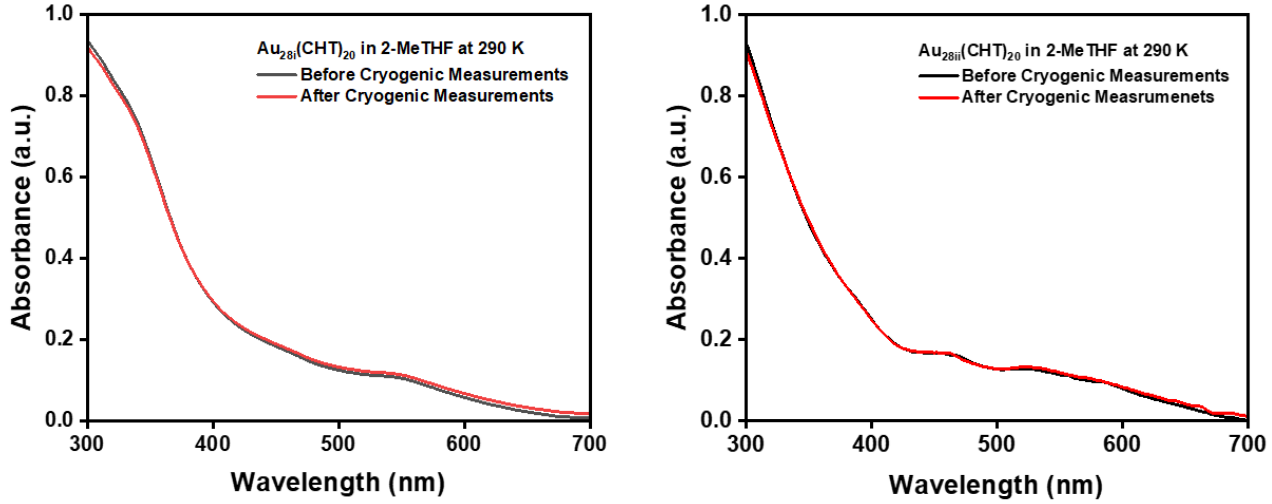

Figure S2. Stability evaluation of (a) **Au_{28i}(CHT)$_{20}$** and (b) **Au_{28ii}(CHT)$_{20}$** in solution by UV-vis absorption spectral comparison before and after cryogenic measurements (the two spectra are almost identical and superimposable).

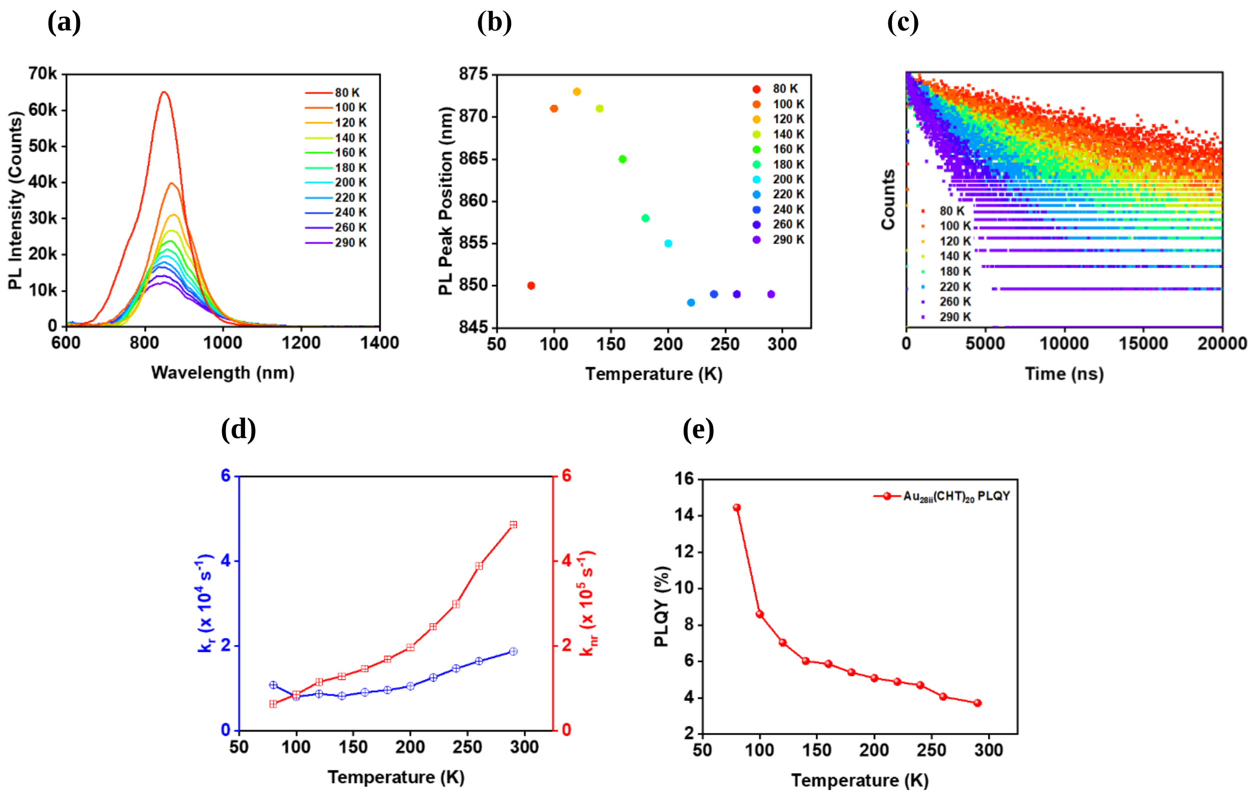

Figure S3. Temperature-dependent data for **$Au_{28ii}(CHT)_{20}$** NC: (a) Photoluminescence (PL) spectra of in 2-methyl-THF, varying the temperature from 290 K to 80 K. (b) Plot of the PL peak position against temperature for the temperature-dependent PL spectra within the temperature range of 290 K to 80 K. (c) PL decay profiles at selected temperatures. (d) Plot of radiative decay rate constants (depicted in blue) and nonradiative decay rate constants (depicted in red) spanning from 80 K to 290 K. (e) Temperature-dependent PL quantum yields (in 2-methyl-THF).

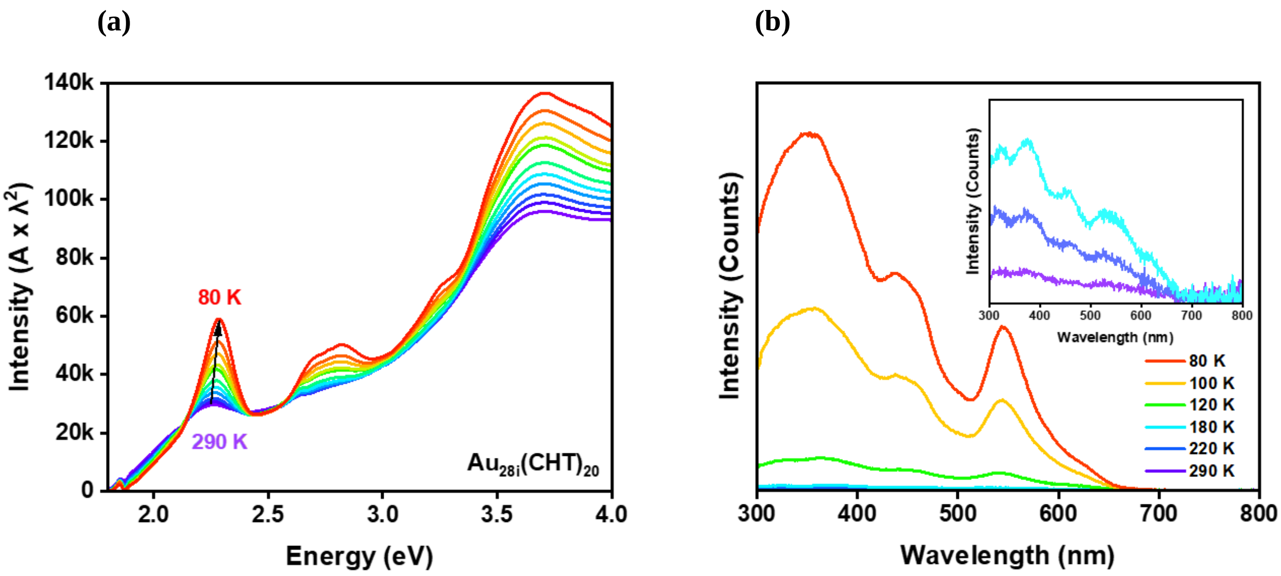

Figure S4. (a) Temperature-dependent UV-vis absorption spectra on the photon energy scale and (b) PL excitation (PLE) spectra of **$Au_{28i}(CHT)_{20}$** in 2-MeTHF (solvent for clear 'glass' formation at cryogenic temperatures). The inset in panel b shows zoom-in PLE spectra at 290 K, 220 K and 180 K. For PLE measurements: excitation slit width 8 nm, and emission slit width 8 nm, PL emission wavelength used for **$Au_{28i}(CHT)_{20}$** is 820 nm.

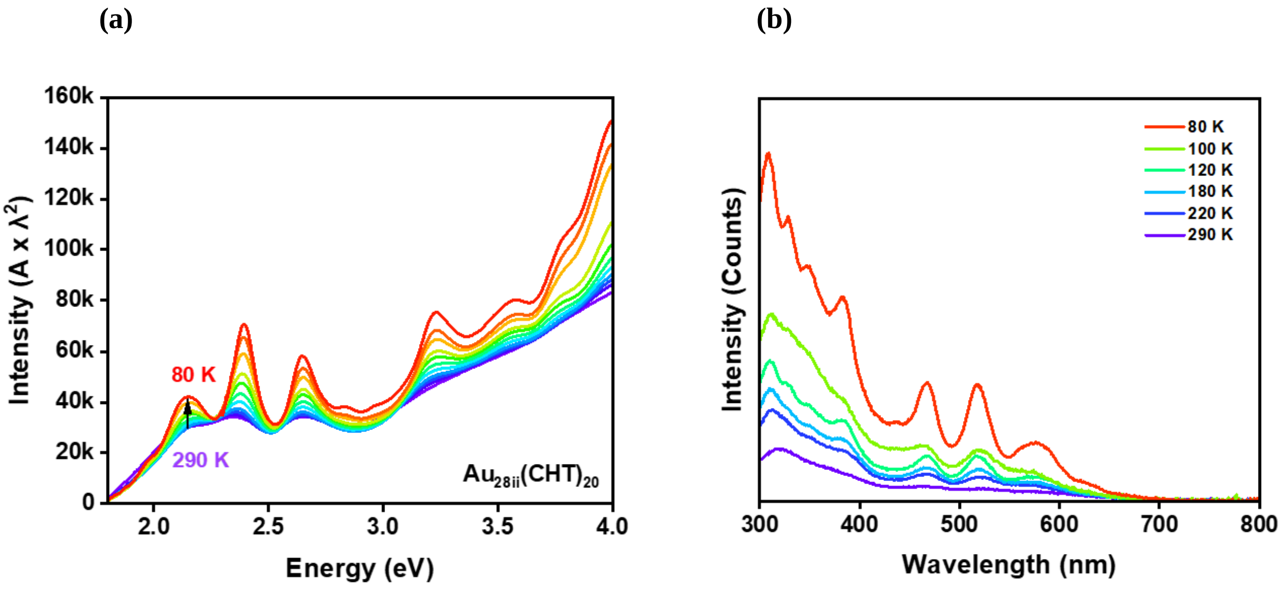

Figure S5. (a) Temperature-dependent UV-vis absorption spectra on the photon energy scale and (b) PLE spectra of **$Au_{28ii}(CHT)_{20}$** in 2-MeTHF. For PLE measurements: excitation slit width 8 nm, and emission slit width 8 nm, PL emission wavelength used for **$Au_{28ii}(CHT)_{20}$** is 850 nm.

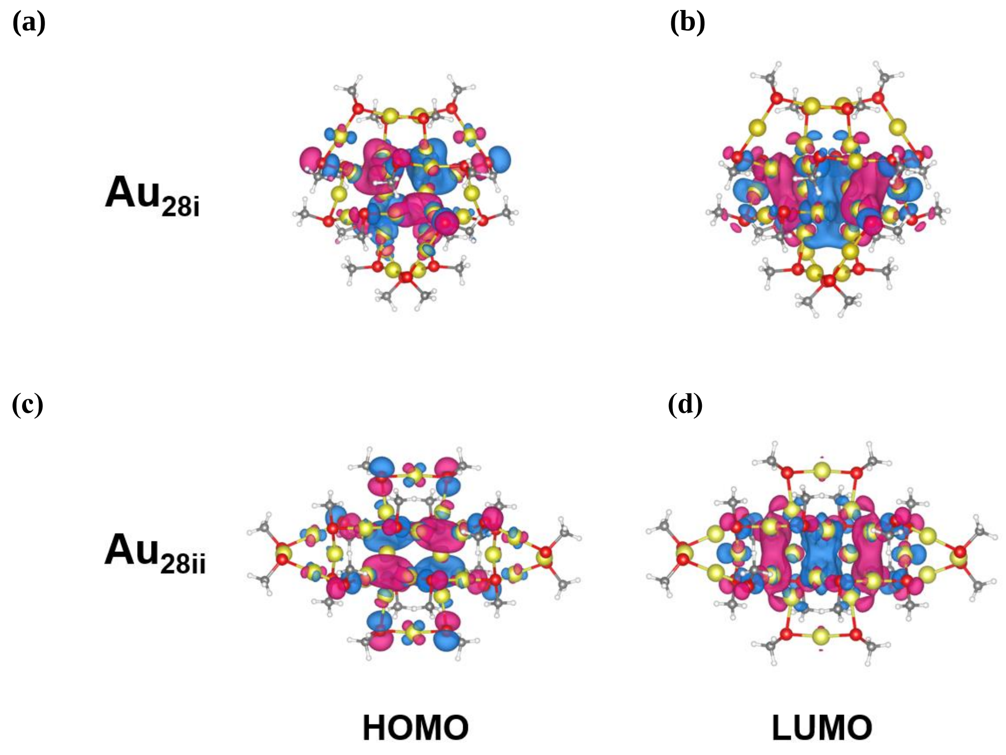

Figure S6. The isosurfaces of frontier orbitals of (a, b) **Au$_{28i}$** and (c, d) **Au$_{28ii}$**.

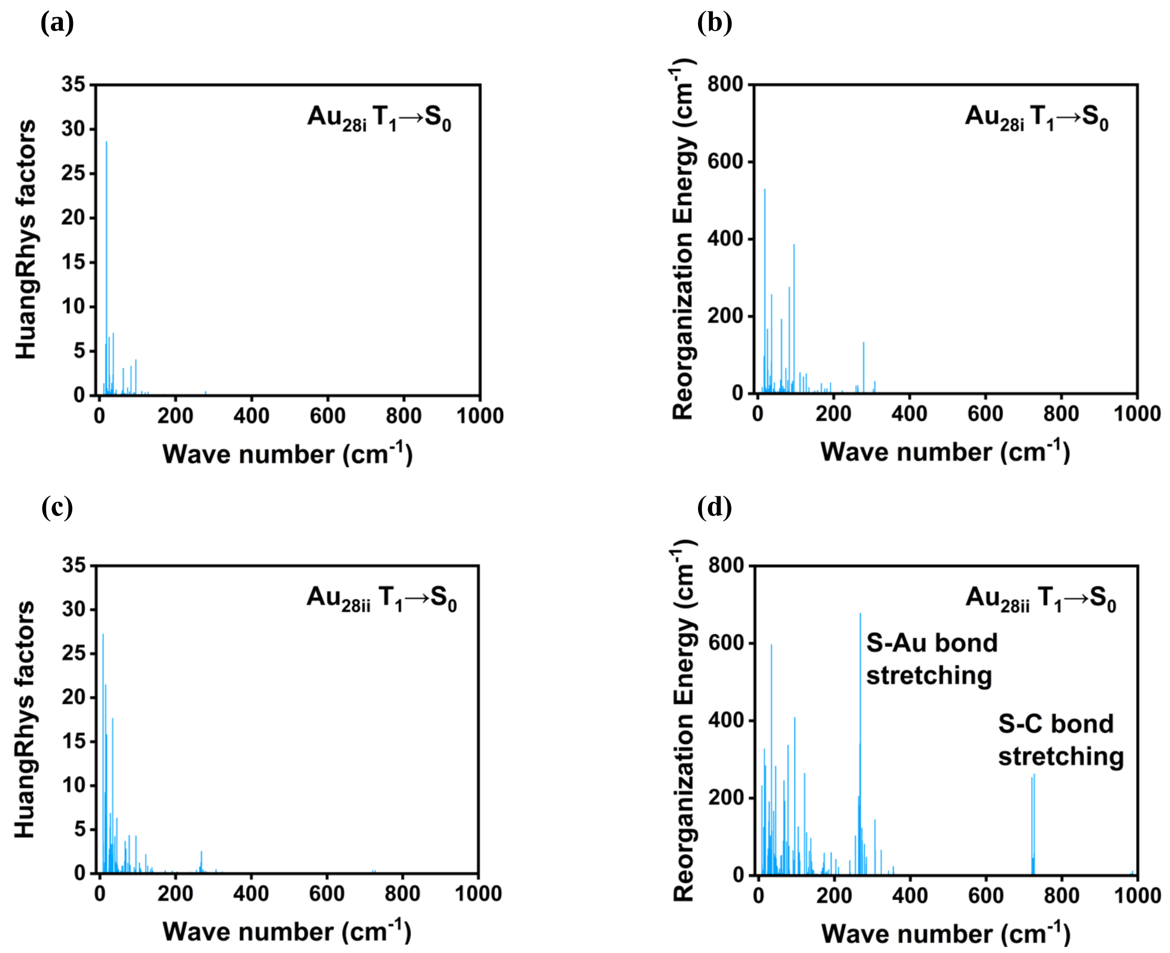

Figure S7. Huang-Rhys factors and reorganization energies of each normal mode for (a, b) $\mathbf{Au_{28i}}$ and (c, d) $\mathbf{Au_{28ii}}$.

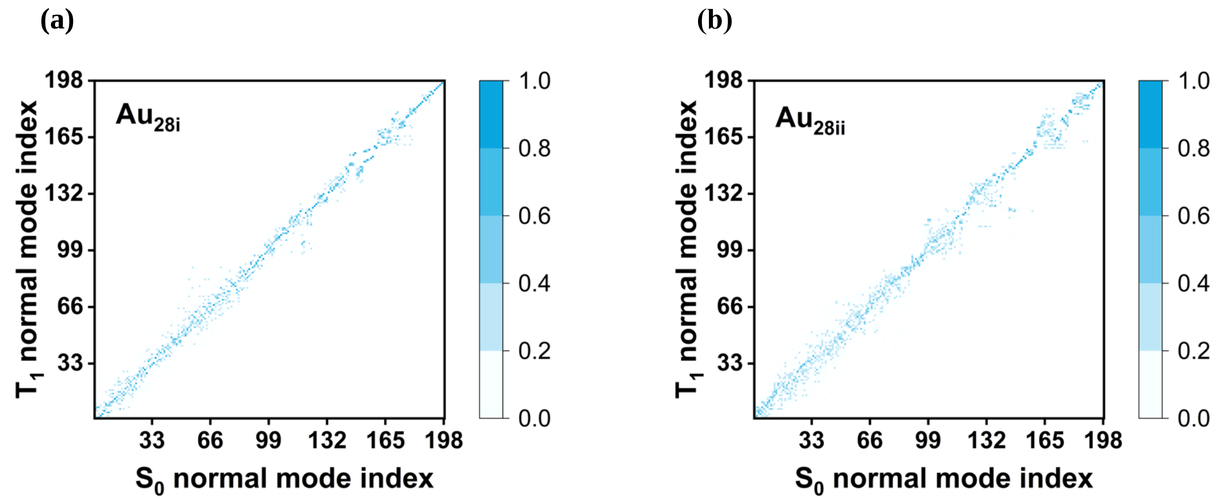

Figure S8. Duschinsky rotation matrix of (a) $\mathbf{Au_{28i}}$ and (b) $\mathbf{Au_{28ii}}$ between T_1 and S_0 normal modes in the low-frequency region (below 400 cm^{-1}).

Table S1. The lifetime values from fitting of temperature-dependent PL decays, percentages (%), average lifetimes, and fitting accuracy (χ^2) for the two Isomeric $Au_{28}(SR)_{20}$ NCs under N_2 atmosphere [a]

Temperature (K)	Nanoclusters				
	$Au_{28i}(CHT)_{20}$				$Au_{28ii}(CHT)_{20}$
	τ_1 (ns)	τ_2 (ns)	τ_{av} (ns)	χ^2	sole τ (ns)
290	256.1 (27.1%)	1434.9 (72.9%)	1115.7	1.0	1981.3 (100%)
260	92.8 (9.0%)	1505.8 (91.0%)	1379.0	1.0	2469.4 (100%)
240	86.9 (10.0%)	1785.5 (90.0%)	1616.2	1.0	3187.2 (100%)
220	104.4 (13.0%)	2055.9 (86.9%)	1801.0	1.0	3872.5 (100%)
200	171.3 (17.7%)	2363.6 (82.3%)	1975.6	1.0	4819.6 (100%)
180	301.6 (21.1%)	2717.7 (78.9%)	2207.4	1.0	5600.2 (100%)
160	437.7 (21.1%)	3088.0 (78.9%)	2528.5	1.0	6430.1 (100%)
140	350.5 (13.2%)	3368.0 (86.8%)	2970.0	1.1	7280.0 (100%)
120	612.4 (13.1%)	4334.5 (86.9%)	3846.9	1.1	8042.6 (100%)
100	9.8 (0.03%)	12466.0 (99.97%)	12462.2	1.0	10603.8 (100%)
80	-	16583.7 (100 %)	16583.7	1.0	13315.7 (100%)

[a]Solvent used: 2-methyl-tetrahydrofuran.

Reference:

(s1) Xia, N.; Yuan, J.; Liao, L.; Zhang, W.; Li, J.; Deng, H.; Yang, J.; Wu, Z. Structural Oscillation Revealed in Gold Nanoparticles. *J. Am. Chem. Soc*. **2020**, *142*, 12140–12145. https://doi.org/10.1021/jacs.0c02117.

(s2) Das, A.; Li, T.; Nobusada, K.; Zeng, C.; Rosi, N. L.; Jin, R. Nonsuperatomic $[Au_{23}(SC_6H_{11})_{16}]^-$ Nanocluster Featuring Bipyramidal Au_{15} Kernel and Trimeric $Au_3(SR)_4$ Motif. *J. Am. Chem. Soc.* **2013**, *135*, 18264–18267. https://doi.org/10.1021/ja409177s.

(s3) Neese, F., The ORCA program system. *WIREs Comput. Mol. Sci.* **2011**, *2*, 73–78. https://doi.org/10.1002/wcms.81

(s4) Neese, F., Software update: The ORCA program system—Version 5.0. *WIREs Comput. Mol. Sci.* **2022**, *12*, e1606. https://doi.org/10.1002/wcms.1606

(s5) Adamo, C.; Barone, V., Toward reliable density functional methods without adjustable parameters: The PBE0 model. *J. Chem. Phys.* **1999**, *110*, 6158–6170. https://doi.org/10.1063/1.478522

(s6) Schäfer, A.; Horn, H.; Ahlrichs, R., Fully optimized contracted Gaussian basis sets for atoms Li to Kr. *J. Chem. Phys.* **1992**, *97*, 2571–2577. https://doi.org/10.1063/1.463096

(s7) Weigend, F., Accurate Coulomb-fitting basis sets for H to Rn. *Phys. Chem. Chem. Phys.* **2006**, *8*, 1057–1065. https://doi.org/10.1039/B515623H

(s8) Frisch, M. J.; Trucks, G. W.; Schlegel, H. B.; Scuseria, G. E.; Robb, M. A.; Cheeseman, J. R.; Scalmani, G.; Barone, V.; Petersson, G. A.; Nakatsuji, H.; Li, X.; Caricato, M.; Marenich, A. V.; Bloino, J.; Janesko, B. G.; Gomperts, R.; Mennucci, B.; Hratchian, H. P.; Ortiz, J. V.; Izmaylov, A. F.; Sonnenberg, J. L.; Williams; Ding, F.; Lipparini, F.; Egidi, F.; Goings, J.; Peng, B.; Petrone, A.; Henderson, T.; Ranasinghe, D.; Zakrzewski, V. G.; Gao, J.; Rega, N.; Zheng, G.; Liang, W.; Hada, M.; Ehara, M.; Toyota, K.; Fukuda, R.; Hasegawa, J.; Ishida, M.; Nakajima, T.; Honda, Y.; Kitao, O.; Nakai, H.; Vreven, T.; Throssell, K.; Montgomery Jr., J. A.; Peralta, J. E.; Ogliaro, F.; Bearpark, M. J.; Heyd, J. J.; Brothers, E. N.; Kudin, K. N.; Staroverov, V. N.; Keith, T. A.; Kobayashi, R.; Normand, J.; Raghavachari, K.; Rendell, A. P.; Burant, J. C.; Iyengar, S. S.; Tomasi, J.; Cossi, M.; Millam, J. M.; Klene, M.; Adamo, C.; Cammi, R.; Ochterski, J. W.; Martin, R. L.; Morokuma, K.; Farkas, O.; Foresman, J. B.; Fox, D. J. Gaussian 16 Rev. C.01, Wallingford, CT, **2016**.

(s9) F. Santoro, FCclasses: A Fortran 77 Code; **2008**. (Available via the Internet at http://www.pi.iccom.cnr.it/Fcclasses; Last Accessed 16/08/2022).

(s10) Humeniuk, A.; Buzancic, M.; Hoche, J.; Cerezo, J.; Mitric, R.; Santoro, F.; Bonacic-Koutecky, V., Predicting fluorescence quantum yields for molecules in solution: A critical assessment of the harmonic approximation and the choice of the lineshape function. *J. Chem. Phys.* **2020**, *152*, 054107. https://doi.org/10.1063/1.5143212

(s11) Liu, Y.; Aranda, D.; Santoro, F., A computational study of the vibronic effects on the electronic spectra and the photophysics of aza[7]helicene. *Phys. Chem. Chem. Phys.* **2021**, *23*, 16551–16563. https://doi.org/10.1039/D1CP00822F
